# Unveiling the AcSirt2‐FOXO‐Mitophagy Axis: Insights Into Mitochondrial Quality Control and Delayed Aging in *Apis cerana*

**DOI:** 10.1111/acel.70645

**Published:** 2026-07-26

**Authors:** Qiang Ma, Zhengang Ma, Tingyue Huang, Qianmin Hai, Xiaoqun Dang, Jinshan Xu, Jialing Bao, Zachary Y. Huang, Zeyang Zhou

**Affiliations:** ^1^ Key Laboratory of Pollinator Resources Conservation and Utilization of the Upper Yangtze River, Ministry of Agriculture and Rural Affairs Chongqing Normal University Chongqing China; ^2^ Natural Drug Intervention in Aging Key Laboratory of Chongqing Education Commission, Department of Basic Medicine Chongqing Three Gorges Medical College Chongqing China; ^3^ Chongqing Key Laboratory of Vector Control and Utilization, College of Life Sciences Chongqing Normal University Chongqing China; ^4^ State Key Laboratory of Resource Insects Southwest University Chongqing China; ^5^ Department of Entomology Michigan State University East Lansing Michigan USA

**Keywords:** aging, *Apis cerana*, FOXO, mitochondrial dynamics, mitophagy, Sirt2

## Abstract

Cellular senescence is closely associated with mitochondrial dysfunction. Sirtuin 2 (Sirt2), a member of the Sirtuin deacetylases family, plays a pivotal role in regulating energy metabolism and aging in mammals. However, its function in social insect aging remains unclear. Here, using the Eastern honey bee ( *Apis cerana* ) as a model, we demonstrate that the age‐related downregulation of *A. cerana* Sirt2 (*AcSirt2*) in brain tissue is coupled with progressive mitochondrial damage, reactive oxygen species (ROS) accumulation, and a biphasic change in autophagy activity. Conversely, overexpression of AcSirt2 alleviates cellular senescence by promoting mitochondrial fusion/fission balance (via Mfn1, Mfn2, and Drp1), activating the PINK1/Parkin‐mediated mitophagy pathway, improving mitochondrial integrity, reducing oxidative stress, and enhancing ATP production. In vivo, AcSirt2 knockdown shortens honey bee lifespan and impairs locomotor ability, whereas its activation reverses these aging phenotypes. Furthermore, we show that AcSirt2 interacts with the transcription factor FOXO and mediates its deacetylation. This study reveals for the first time that the AcSirt2‐FOXO‐mitophagy axis delays aging by maintaining mitochondrial homeostasis in a social insect, providing novel insights into the development of anti‐aging strategies and the promotion of healthy beekeeping.

## Graphical abstract

Age‐related downregulation of AcSirt2 impairs mitochondrial homeostasis in old bees. AcSirt2‐mediated deacetylation of FOXO enhances transcriptional activity, upregulating antioxidant enzymes (SOD, CAT) and promoting PINK1/Parkin‐mediated mitophagy. This pathway facilitates autophagosome formation and clearance of damaged mitochondria, reducing ROS accumulation. Restored mitochondrial integrity enhances TCA cycle enzyme activities (IDH, α‐KGDH) and ATPase, boosting ATP production and cell viability, ultimately delaying aging.

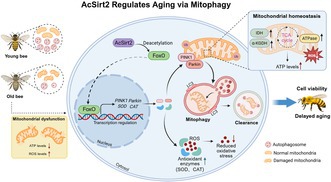

## 1 Introduction

Aging is a physiological process in all organisms, characterized by the accumulation of molecular and cellular damages and thus progressive functional decline (Guo et al. 2022). During this complex process, imbalance of mitochondrial homeostasis is recognized as one of the core cellular events. Mitochondrial homeostasis plays crucial roles in regulating metabolism, development, oxidative stress levels, and is implicated in both physiological health and pathological processes (Jadeja et al. 2021; Lopez‐Otin et al. 2023; Li et al. 2024). Therefore, in‐depth exploration of the molecular regulatory mechanisms underlying mitochondrial homeostasis and quality control holds significance for developing interventions to delay aging and age‐related diseases.

Social insects such as honey bees (genus *Apis*), including the Eastern honey bee ( *Apis cerana* ) and the Western honey bee ( *Apis mellifera* ), provide a unique model system for studying aging mechanisms in a natural social context, due to their distinct age‐related division of labor and plastic lifespan (Keller and Jemielity 2006; Münch et al. 2013; Speth et al. 2015). In these species, young honey bees are primarily responsible for in‐nest nursing, middle‐aged bees shift to foraging outside the nest, and older bees exhibit decreased behavioral flexibility due to physiological decline (Yao et al. 2025). This series of age‐related behavioral transitions is accompanied by progressive deterioration of mitochondrial function and accumulation of oxidative damage in brain tissue, presenting molecular and cellular features highly similar to those of mammalian aging (Alsolami 2023; Zhang et al. 2025). Notably, as the core organ regulating behavior and cognitive function, the honey bee brain undergoes significant mitochondrial structural damage and reduced autophagic activity during aging, directly affecting the survival and reproduction of the colony (Margotta et al. 2012; Monroy Kuhn and Korb 2016; Ma et al. 2025). However, the underlying molecular mechanisms regulating this process remain to be systematically elucidated.

The maintenance of mitochondrial dynamic balance and quality control such as mitophagy is essential for cell survival (Sedlackova and Korolchuk 2019; Kowluru and Alka 2023). It's reported that the mitochondrial fusion proteins (Mfn1, Mfn2) and fission protein (Drp1) coordinately regulate mitochondrial network morphology, while the PINK1/Parkin‐mediated mitophagy pathway is responsible for clearing damaged mitochondria. Dysfunction of either process accelerates mitochondrial impairment and aging (Leduc‐Gaudet et al. 2021; Wang et al. 2024). Given the central role of mitochondrial quality control in aging, identifying key molecular regulators of these processes is essential. In recent years, the Sirtuin family of proteins has attracted considerable attention due to their roles in aging regulation, energy metabolism, oxidative stress responses, autophagy regulation and so on (Xu and Wan 2023; Yao et al. 2023; Baeken 2024; Lagunas‐Rangel 2025). Sirtuins are a class of highly conserved NAD^+^‐dependent deacetylases found in organisms across all domains of life, from archaea to mammals. Their enzymatic activity depends on the cofactor β‐nicotinamide adenine dinucleotide (NAD^+^), making them key targets in calorie restriction strategies for delaying aging. Known as the “longevity protein family,” sirtuins play roles in regulating lifespan across multiple species. The human sirtuin family (SIRT1‐7) all possess an NAD^+^‐binding site and a relatively conserved catalytic domain, yet differ in subcellular localization and activity (Haigis and Sinclair 2010). Among them, Sirt2, which is widely expressed in the cytoplasm and nucleus, is well‐studied in mammalian organisms. The key mechanism of Sirt2 regulating aging is by deacetylating the Forkhead box O (FOXO) transcription factor therefore to enhance mitochondrial function and metabolic homeostasis (Liu et al. 2017; Zhu et al. 2022). In yeast, homologous Sir2 genes have also been reported to improve aging phenotypes by remodeling mitochondrial networks (Wang et al. 2019). However, the function of Sirt2 in insects, particularly its mechanism of action in regulating mitochondrial homeostasis and lifespan in honey bees, remains poorly understood.

In *Drosophila melanogaster* , Parkin‐mediated mitophagy and the FOXO signaling pathway cooperatively regulate lifespan (Rana et al. 2013). Notably, SIRT2 regulates mitochondrial dynamics through the deacetylation of FOXO in mammals (Wang and Tong 2009), highlighting its potential role in maintaining aging‐related mitochondrial homeostasis via regulating Parkin‐mediated mitophagy and FOXO in insects. Analysis of previously published transcriptomic data from the brain tissue of *A. cerana* (Ma et al. 2025) revealed that the expression of sirtuin pathway‐related genes exhibits significant age‐associated changes, and differentially expressed genes are significantly enriched in the FOXO signaling pathway and mitophagy pathway. This provides important clues for exploring the biological function of AcSirt2 in honey bee aging, and whether AcSirt2 and FOXO have direct interactions or cooperatively regulate honey bee mitochondrial dynamics and aging.

Given the established importance of mitochondrial homeostasis in aging and prior transcriptomic clues linking the Sirtuin pathway to aging in *A. cerana* , we hypothesized that AcSirt2 serves as a key regulator of aging by deacetylating FOXO and activating PINK1/Parkin‐mediated mitophagy to preserve mitochondrial quality. To test this hypothesis, the present study employs *A. cerana* as a model to systematically investigate the function and underlying molecular mechanisms of AcSirt2. We first characterize age‐dependent changes in mitochondrial dynamics, autophagic activity, and AcSirt2 expression. Then, its regulatory effects on honey bee lifespan, locomotor performance, and brain mitochondrial integrity were verified via genetic and pharmacological approaches. Furthermore, its capacity to mitigate oxidative stress‐induced cellular senescence and improve mitochondrial quality in vitro was assessed. Finally, the molecular mechanism by which AcSirt2 interacts with the FOXO‐PINK1/Parkin axis was dissected. This study aims to unveil a novel Sirt2‐mediated regulatory mechanism in honey bee aging, thereby providing not only a fresh molecular perspective for insect aging research but also a potential target for developing anti‐aging strategies.

## 2 Results

### 2.1 Brain Mitochondrial Dysfunction and Age‐Dependent Decline in Mitophagy Capacity in *A. cerana*

**FIGURE 1:**
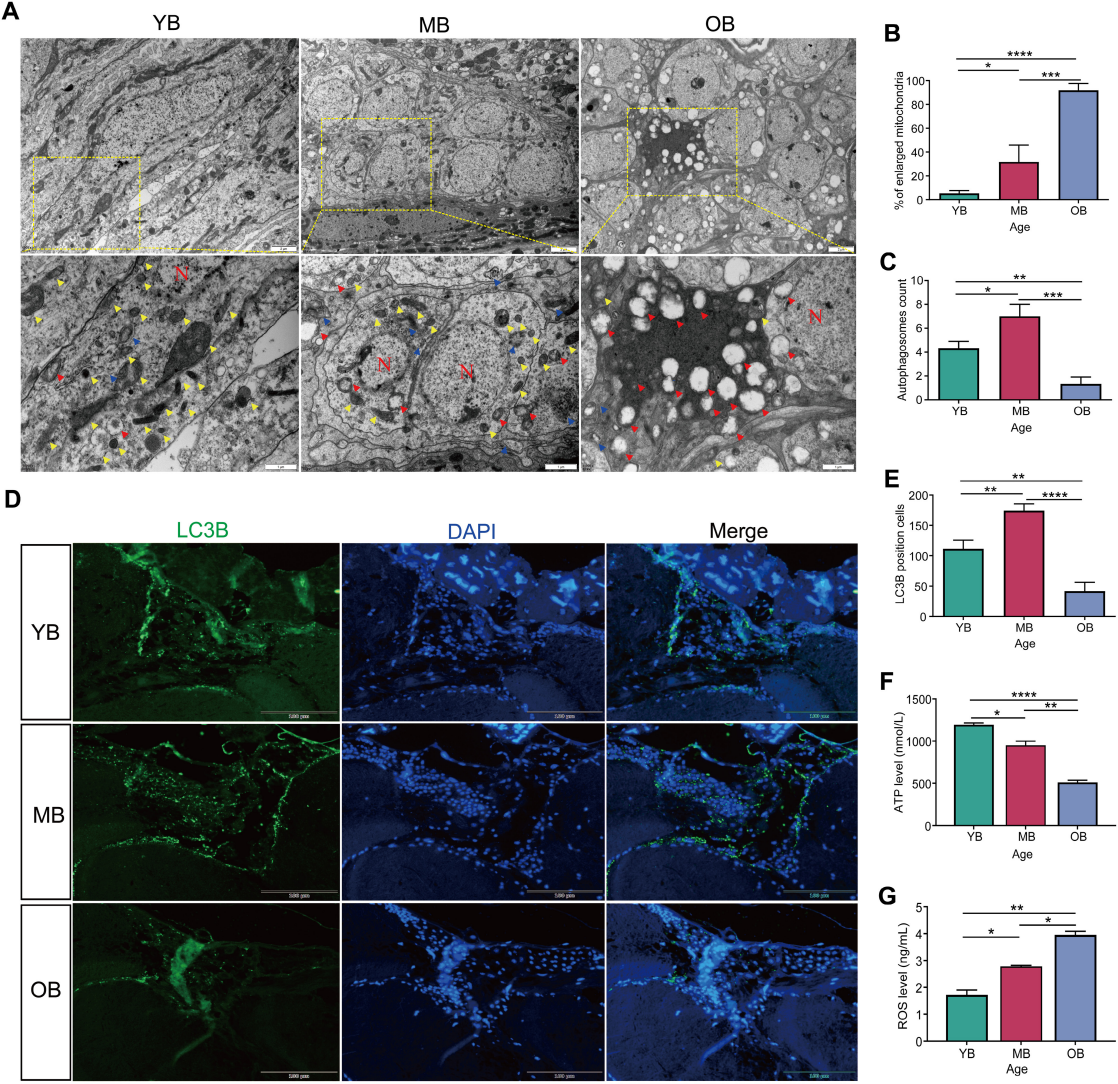
Age‐dependent changes in mitochondrial ultrastructure, autophagic activity, and energy metabolism in the brains of *A. cerana* . (A) TEM images of brain tissue ultrastructure in worker bees of different ages (*n* = 3; five fields of view per bee). Yellow arrows indicate normal mitochondria, red arrows indicate damaged mitochondria, blue arrows indicate autophagosomes, and N denotes the nucleus. (B) Quantitative analysis of mitochondrial damage. The extent of damage was assessed by calculating the percentage of mitochondria with abnormal morphology per field of view (*n =* 3; five fields per bee). (C) Quantification of autophagosomes. The number of autophagosomes was counted in five randomly selected fields (*n =* 3). (D) Merged immunofluorescence staining of LC3B and DAPI in brain tissue sections of worker bees of different ages (*n* = 3; five fields per bee). DAPI (blue) stains nuclei, and punctate LC3B signals (green) indicate autophagosomes, with the merged image showing the autophagosome distribution and their relative localization to nuclei in brain cells. (E) Percentage of LC3B‐positive cells. Cells displaying distinct LC3B puncta were counted in five random fields per sample (*n* = 3). (F) ATP content in brains of worker bees of different ages (*n* = 3). (G) ROS levels in brains of worker bees of different ages (*n* = 3). Data in (B, C, E–G) are presented as mean ± SEM. Statistical significance in (B, C, E–G) was determined by one‐way ANOVA [*F* (2, 6)] with Tukey's post hoc test: *p < 0.05, **p < 0.01, ***p < 0.001, ****p < 0.0001.

The ultrastructural changes of brain mitochondria in young bees (YBs), middle‐aged bees (MBs), and old bees (OBs) by TEM were first investigated. Mitochondria in YBs exhibited intact structures with clear and visible cristae and the lowest mitochondrial damage rate (Figure 1A,B; damage rate: 5.4% ± 1.9%). MBs displayed moderate mitochondrial damage (Figure 1B; damage rate: 31.7% ± 8.9%). For autophagosomes, their number in MBs increased by 1.6‐fold compared to YBs (Figure 1C; *p* < 0.05). In contrast, OBs exhibited severe mitochondrial degenerative changes (Figure 1B; damage rate: 86.3% ± 3.6%), including extensive mitochondrial swelling, vacuolization, complete loss of cristae, and disintegration of membrane structures (Figure 1A). Notably, the number of autophagosomes in OBs was significantly reduced compared to both YBs and MBs (Figure 1C; OBs vs. YBs, *p* < 0.01; OBs vs. MBs, *p* < 0.001).

Consistent with the TEM observations, immunofluorescent staining of the autophagic marker LC3B illustrated the most intense fluorescence signals in the brains of MBs, compared to the markedly weakened signals in OBs (Figure 1D). The percentage of LC3B‐positive cells was also significantly lower in the OBs compared to YBs and MBs (Figure 1E; *p* < 0.01, *p* < 0.0001), suggesting an impairment of autophagic flux in OBs.

Similarly, biochemical assays of brain tissue showed an age‐dependent decrease in ATP levels (Figure 1F) while a significant increase in ROS levels with aging (Figure 1G). Collectively, these results indicate that mitochondrial function in the brains of *A. cerana* progressively declines with aging, accompanied by a significant reduction in autophagic capacity.

### 2.2 AcSirt2 Expression Declined With Age and Correlated With Mitochondrial and Autophagic Dysfunction

**FIGURE 2:**
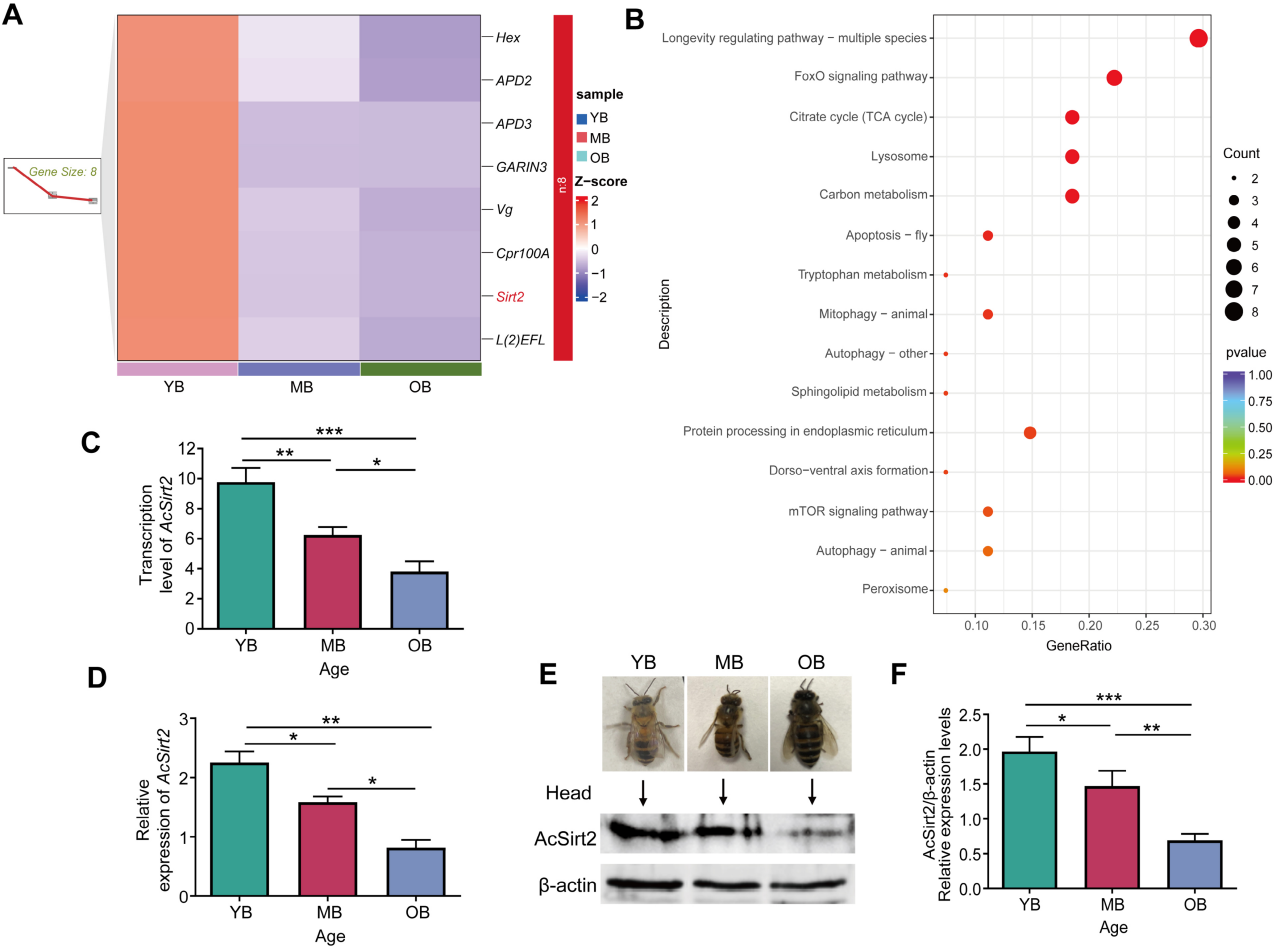
Age‐associated downregulation of *AcSirt2* and its association with aging‐related pathways. (A) Heatmap of gene expression profiles from transcriptomic sequencing of head tissues in worker bees across different ages (*n =* 3 per age group; each replicate pooled from three bee heads). The color gradient from red to blue indicates high to low expression levels. (B) KEGG pathway enrichment analysis of DEGs (|log_2_FC| ≥ 1, FDR < 0.05). Bubble size corresponds to the number of enriched genes, and color represents the significance of enrichment. (C) Transcriptional level of *AcSirt2* in the brains of worker bees from different age groups (*n* = 3). (D) RT‐qPCR validation of *AcSirt2* mRNA expression levels across age groups (*n* = 3). (E) Western blot analysis of AcSirt2 protein expression in head tissues (*n* = 3); β‐Actin served as the loading control. (F) Quantification of relative AcSirt2 protein expression (*n* = 3). Data in (C, D, F) are presented as mean ± SEM. Statistical significance in (C, D, F) was determined by one‐way ANOVA [*F* (2, 6)] with Tukey's post hoc test: *p < 0.05, **p < 0.01, ***p < 0.001.

Based on the previously observed age‐related mitochondrial damage and autophagic dysfunction, differentially expressed genes (DEGs) in the brains of worker bees across different age groups were screened through transcriptomic analysis and identified a set of genes closely associated with the aging process (Figure 2A). KEGG pathway enrichment analysis revealed that these DEGs were significantly enriched in pathways such as “Longevity regulating pathway”, “FOXO signaling pathway”, “TCA cycle”, and “Mitophagy” (Table S1 and Figure 2B), suggesting that mitochondrial quality was criticalin brain aging of honey bees. For complete transparency, Table S1 lists all KEGG pathways obtained from the analysis, including those with adjusted *p*‐values (*p*_adj_) > 0.05, while Figure 2B displays the top 15 enriched pathways ranked by *p*‐value, the majority of which reached statistical significance (*p*_adj_ < 0.05). During the screening process, *Sirt2* was identified as a key candidate gene due to its known role in lifespan regulation in model organisms and its significant transcriptional downregulation in the brains of both MBs and OBs (Figure 2C).

To investigate the expression patterns of AcSirt2 in honeybee brains, RT‐qPCR and Western blot were employed to detect its mRNA and protein levels, respectively. Transcription of AcSirt2 gradually decreased with age (Figure 2D), and its protein expression also exhibited a consistent age‐dependent reduction (Figure 2E,F). Together, these data confirmed a negative correlation between AcSirt2 expression and the brain aging process in *A. cerana* . Combined with its evolutionary conservation and the observed declines in mitochondrial function and autophagic activity, these findings suggested that AcSirt2 may influence honey bee lifespan by regulating mitochondrial homeostasis and autophagic processes, thereby prompting us to perform subsequent functional studies.

### 2.3 AcSirt2 Alleviates Oxidative Stress‐Induced Cellular Senescence and Maintains Mitochondrial Homeostasis by Regulating Mitochondrial Dynamics

**FIGURE 3:**
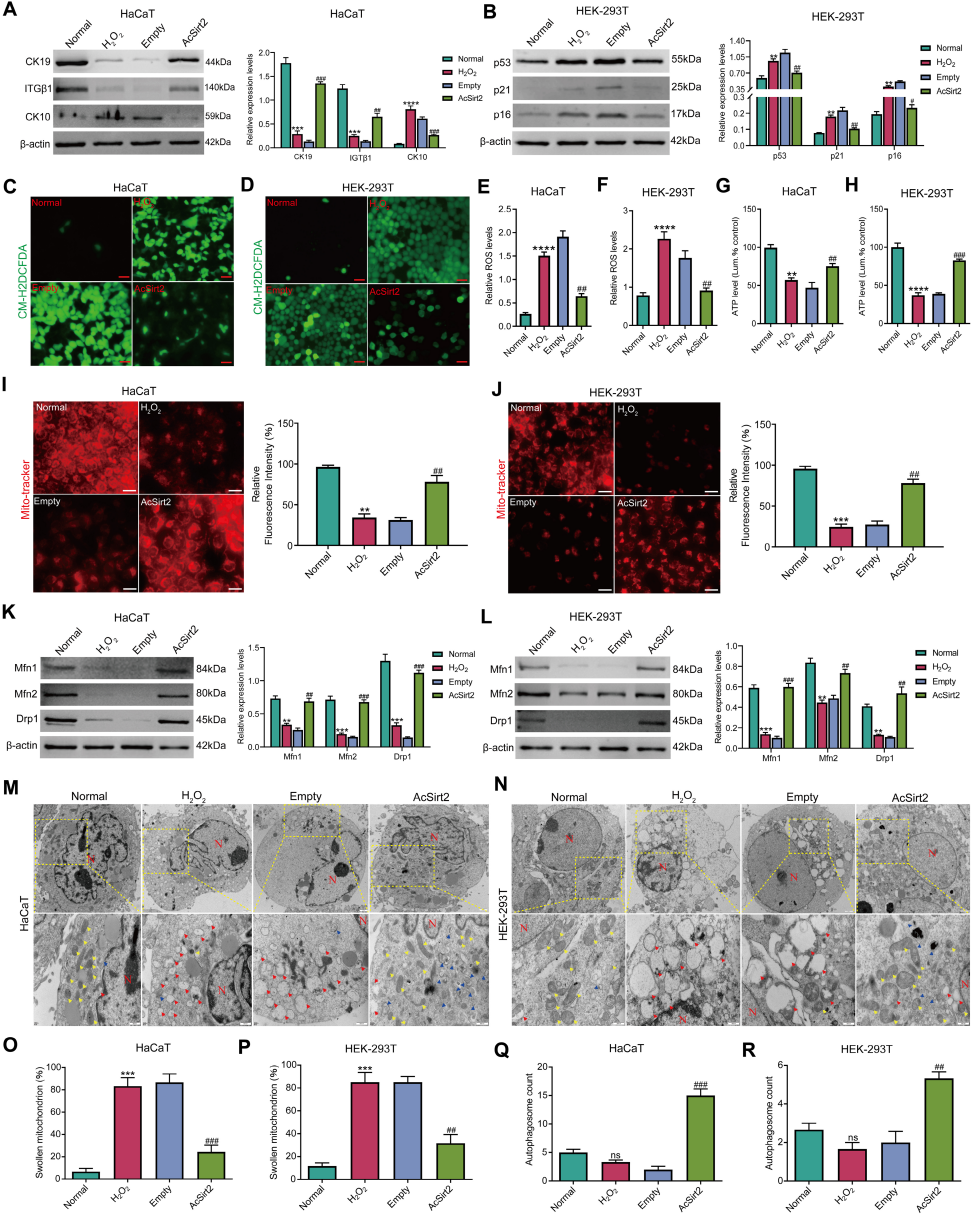
AcSirt2 overexpression alleviates oxidative stress‐induced senescence and maintains mitochondrial dynamics and quality in mammalian cells. (A) Western blot analysis of the protein expression levels of the cellular markers CK19, β1 integrin, and CK10 in HaCaT cells (*n* = 3). (B) Western blot analysis of the senescence markers p53, p21, and p16 in HEK‐293T cells (*n* = 3). (C, D) Representative fluorescence images of intracellular ROS detected by CM‐H2DCFDA probe in HaCaT (C) and HEK‐293T (D) cells. Scale bar: 50 μm (*n* = 3). (E, F) Intracellular ROS levels measured by fluorometry in HaCaT (E) and HEK‐293T (F) cells (*n* = 3). (G, H) Intracellular ATP levels in HaCaT (G) and HEK‐293T (H) cells detected by CellTiter‐Glo assay (*n* = 3). (I, J) Mito‐Tracker Red staining assessed mitochondrial network integrity and relative fluorescence intensity in HaCaT (I) and HEK‐293T (J) cells. Scale bar: 20 μm (*n* = 3). (K, L) Western blot analysis of Mfn1, Mfn2, and Drp1 protein expression levels in HaCaT (K) and HEK‐293T (L) cells (*n* = 3). (M, N) TEM images of mitochondrial ultrastructure in HaCaT (M) and HEK‐293T (N) cells. Scale bar: 500 nm (*n* = 3; five fields per sample). Yellow arrows indicate normal mitochondria; red arrows indicate damaged mitochondria; blue arrows indicate autophagosomes. (O, P) Quantification of mitochondrial damage rate in HaCaT (O) and HEK‐293T (P) cells (*n* = 3; five fields per sample). (Q, R) Quantification of autophagosome numbers in HaCaT (Q) and HEK‐293T (R) cells (*n* = 3; five fields per sample). Data in (A–L, O–R) are presented as mean ± SEM. Statistical significance in (A–L, O–R) was determined by one‐way ANOVA [*F* (3, 8)] with Tukey's post hoc test: Ns, no significant; **p < 0.01, ***p < 0.001, ****p < 0.0001 compared with the normal group; ^#^ *p* < 0.05, ^##^ *p* < 0.01, ^###^ *p* < 0.001 compared with the H_2_O_2_‐treated group. No significant differences were detected between the H_2_O_2_‐treated group and the H_2_O_2_ + Empty vector group (*p* > 0.05).

To investigate the potential anti‐senescence function of AcSirt2, we overexpressed it in mammalian cells. A recombinant expression plasmid, pcDNA3.1‐*AcSirt2*, carrying EGFP or Flag tags was constructed (Figure S1A) and transfected into HaCaT and HEK‐293T cells. Successful expression was confirmed by fluorescence microscopy and Western blotting (Figure S1B–D). In the H_2_O_2_‐induced senescence model, AcSirt2 overexpression significantly enhanced cell viability (Figure S1E,F), and reduced the proportion of SA‐β‐gal‐positive cells (Figure S1G,H). In HaCaT cells, AcSirt2 upregulated epidermal stem cell markers CK19 and β1‐integrin (ITGβ1) and downregulated the terminal differentiation marker CK10 (Figure 3A and Figure S2A–C). In HEK‐293T cells, it suppressed the senescence regulators p53, p21, and p16 (Figure 3B and Figure S2D–F). Furthermore, H_2_O_2_ induction significantly increased ROS fluorescence intensity in both cell lines (Figure 3C,D), while AcSirt2 overexpression effectively reversed the H_2_O_2_‐induced elevation of intracellular ROS levels (*p* < 0.01) (Figure 3E,F). Meanwhile, intracellular ATP levels were significantly increased in both HaCaT and HEK‐293T cells transfected with AcSirt2 (*p* < 0.01, *p* < 0.001) (Figure 3G,H). These results indicate that AcSirt2 alleviates oxidative stress‐triggered cellular senescence and improves energy metabolism in mammalian cells.

Given these anti‐senescence effects, we next investigated whether AcSirt2 protects mitochondrial function. We first examined mitochondrial network morphology using Mito‐Tracker Red staining. In normal cells, a continuous and uniform mitochondrial network was observed. However, after H_2_O_2_ treatment, both HaCaT and HEK‐293T cells exhibited weakened red fluorescence intensity and fragmented distribution (Figure 3I,J), indicating the distortion of mitochondrial morphology. Overexpression of AcSirt2 significantly restored fluorescence intensity and improved network continuity (Figure 3I,J).

Given the close relationship between mitochondrial quality and mitochondrial dynamic balance, we further examined the expression of key regulatory proteins in mitochondrial dynamics. Western blot showed that the expression levels of the fusion proteins Mfn1, Mfn2, and the fission protein Drp1 were significantly reduced in H_2_O_2_‐treated HaCaT and HEK‐293T cells (Figure 3K,L). Interestingly, AcSirt2 overexpression resulted in varying degrees of recovery in the expression of these proteins (Figure 3K,L). In HaCaT cells, protein levels of Mfn1, Mfn2, and Drp1 increased by 2.0‐fold, 3.5‐fold, and 3.4‐fold, respectively, compared to the H_2_O_2_ group (*p* < 0.01, *p* < 0.001, *p* < 0.001, respectively) (Figure 3K). In HEK‐293T cells, the relative expression levels of these three proteins increased by 4.4‐fold, 1.6‐fold, and 4.1‐fold, respectively (*p* < 0.001, *p* < 0.01, *p* < 0.01) (Figure 3L). RT‐qPCR results confirmed that AcSirt2 overexpression significantly upregulated the mRNA levels of *Mfn1*, *Mfn2*, and *Drp1* in both cell lines (Figure S3A–F).

TEM provided ultrastructural evidence supporting these findings. Compared to normal mitochondrial morphology, H_2_O_2_ treatment caused severe swelling, vacuolization, and cristae fragmentation in both HaCaT and HEK‐293T cells, with a significant increase in mitochondrial damage rate (*p* < 0.001). In contrast, AcSirt2 overexpression significantly improved mitochondrial morphology and reduced the damage rate (*p* < 0.001, *p* < 0.01) (Figure 3M–P). Notably, the number of autophagosomes was significantly increased in AcSirt2‐transfected cells (*p* < 0.001, *p* < 0.01) (Figure 3Q,R), suggesting its potential involvement in mitochondrial quality control through activation of mitophagy.

### 2.4 AcSirt2 Activates PINK1/Parkin‐Mediated Mitophagy to Maintain Mitochondrial Homeostasis

**FIGURE 4:**
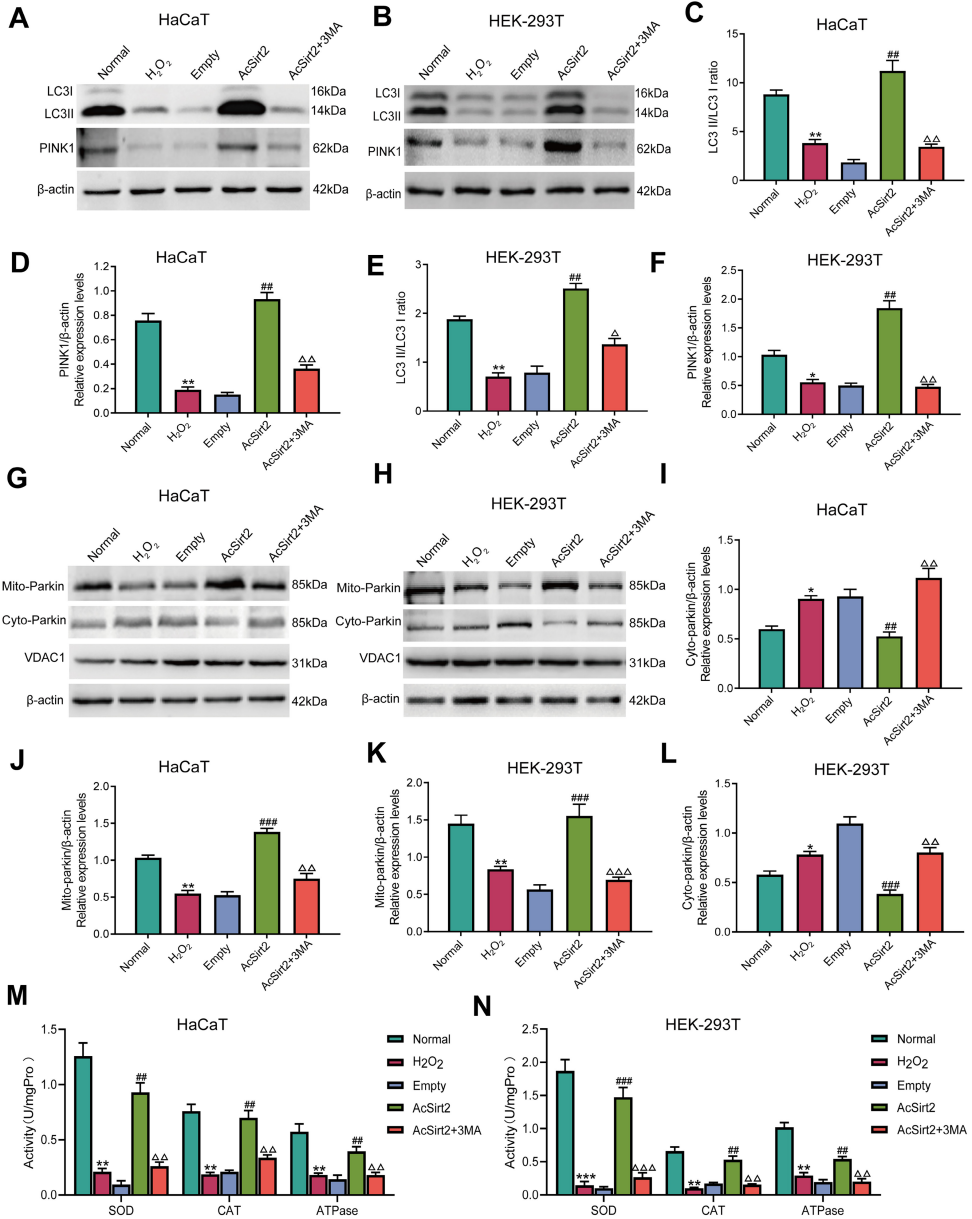
AcSirt2 maintained mitochondrial homeostasis by activating PINK1/Parkin‐mediated mitophagy. (A, B) Western blot analysis of LC3 and PINK1 protein expression in HaCaT (A) and HEK‐293T (B) cells (*n* = 3). (C–F) Quantification of the LC3‐II/LC3‐I ratio and PINK1 protein levels in HaCaT (C, D) and HEK‐293T (E, F) cells (*n* = 3). (G, H) Western blot analysis of Parkin distribution in mitochondrial and cytosolic fractions from HaCaT (G) and HEK‐293T (H) cells (*n* = 3). VDAC1 was used as a mitochondrial loading control. (I–L) Quantification of Mito‐Parkin and Cyto‐Parkin protein levels in HaCaT (I, J) and HEK‐293T (K, L) cells (*n* = 3). (M, N) Activities of SOD, CAT, and ATP synthase in HaCaT (M) and HEK‐293T (N) cells (*n* = 3). 3‐MA was used as an autophagy inhibitor. Data in (C–F, I–N) are presented as mean ± SEM. Statistical significance (C–F, I–N) was determined by one‐way ANOVA [*F* (4, 10)] with Tukey's post hoc test: *p < 0.05, **p < 0.01, ***p < 0.001 compared with the Normal group; ^##^ *p* < 0.01, ^###^ *p* < 0.001 compared with the H_2_O_2_‐treated group; ^Δ^ *p* < 0.05, ^ΔΔ^ *p* < 0.01, ^ΔΔΔ^ *p* < 0.001 compared with the AcSirt2 transfection group.

Compared with the H_2_O_2_‐induced senescence model group, AcSirt2 transfection significantly increased the LC3‐II/LC3‐I ratio and enhanced PINK1 protein expression (Figure 4A,B). In HaCaT cells, the LC3‐II/LC3‐I ratio and PINK1 protein levels were increased by 2.9‐fold and 4.9‐fold, respectively (both *p* < 0.01; Figure 4C,D). In HEK‐293T cells, these values were increased by 3.6‐fold and 3.3‐fold (both *p* < 0.01; Figure 4E,F). The AcSirt2‐induced elevations were markedly suppressed by the mitophagy inhibitor 3‐MA, further confirming the direct regulation function of AcSirt2 on mitophagy (Figure 4A,B). In addition, RT‐qPCR showed that the mRNA levels of *LC3*, *PINK1*, and *Beclin1* were significantly downregulated following H_2_O_2_‐induced senescence but were notably upregulated upon AcSirt2 transfection in both cell lines (Figure S4A–F).

To further investigate the regulatory mechanism of AcSirt2 in the PINK1/Parkin pathway, the subcellular distribution of Parkin was assessed. Western blot of Parkin in mitochondrial (mito‐Parkin) and cytosolic (cyto‐Parkin) fractions was performed, with VDAC1 serving as a marker for successful mitochondrial extraction (Figure 4G,H). H_2_O_2_ treatment significantly decreased mito‐Parkin while increasing cyto‐Parkin levels in both cell lines. AcSirt2 overexpression reversed this distribution pattern (Figure 4G,H). Specifically, in HaCaT cells, mito‐Parkin levels were 2.5‐fold higher, and cyto‐Parkin levels were reduced by 41.9% ± 3.7% compared to the H_2_O_2_ group (*p* < 0.001, *p* < 0.01; Figure 4I,J). In HEK‐293T cells, mito‐Parkin increased by 1.9‐fold (*p* < 0.001), while cyto‐Parkin decreased by 50.9% ± 5.5% (*p* < 0.001; Figure 4K,L).

To determine the necessity of mitophagy in AcSirt2‐mediated regulation of mitochondrial function, we measured the activities of antioxidant enzymes and ATPase. The activities of SOD, CAT, and ATPase were significantly reduced in H_2_O_2_‐induced senescent cells compared to the normal group. AcSirt2 overexpression significantly rescued these activities (*p* < 0.01). Critically, this enhancement was largely abolished by the mitophagy inhibitor 3‐MA (*p* < 0.01), demonstrating that the restoration of mitochondrial function was dependent on mitophagy (Figure 4M,N). Collectively, these results demonstrate that AcSirt2 activates the PINK1/Parkin‐mediated mitophagy pathway to promote the clearance of damaged mitochondria, restore antioxidant enzyme activities, and ATP synthesis capacity, thereby maintaining mitochondrial homeostasis and delaying cellular senescence.

### 2.5 In Vivo Knockdown of AcSirt2 Accelerates Aging and Impairs Motor Function, Whereas Its Activation Extends Lifespan and Enhances Vitality

**FIGURE 5:**
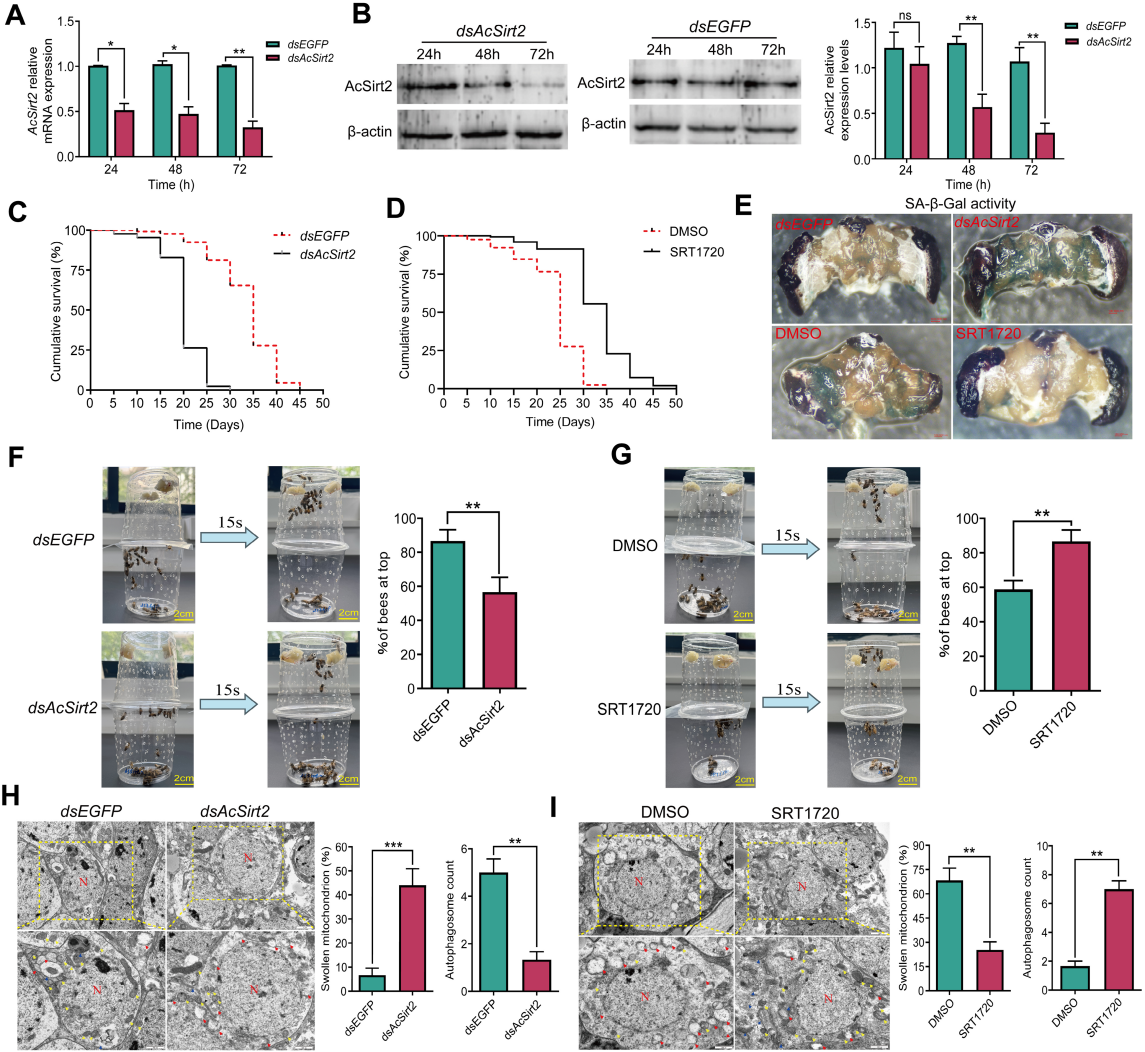
In vivo modulation of AcSirt2 influences aging, motor function, and brain ultrastructure in *A. cerana* . (A) Relative mRNA expression levels of *AcSirt2* in the brains of worker bees at 24, 48, and 72 h after *dsAcSirt2* or *dsEGFP* (control) treatment, determined by RT‐qPCR (*n* = 3; 3 bees per time point per treatment). (B) AcSirt2 protein expression levels in bee brains at 24, 48, and 72 h post *dsAcSirt2* or *dsEGFP* treatment, analyzed by Western blot (*n* = 3; 3 bees per time point per treatment). (C) Survival curves of worker bees continuously fed *dsAcSirt2* or *dsEGFP* (control) from day 7 after eclosion (*N =* 150 per group; 50 bees per cup, 3 biological replicates). (D) Survival curves of worker bees treated with the Sirt2 activator SRT1720 or DMSO (control) from day 7 after eclosion (*N* = 150 per group; 50 bees per cup, 3 biological replicates). (E) Senescence‐associated β‐galactosidase (SA‐β‐gal) staining of worker bee brains from different treatment groups. Blue staining indicates SA‐β‐gal‐positive senescent cells (*n* = 3). (F, G) Motor function tests of worker bees in different treatment groups and the percentage of bees that reached the top feeding platform within 15 s (*N* = 120 per group; 40 bees per cup, 3 biological replicates). (H, I) TEM images of brain cells from worker bees in different treatment groups (scale bar: 500 nm) and quantitative analysis of mitochondrial damage rate and autophagosome number (*n* = 3; five fields per bee). Yellow arrows indicate normal mitochondria; red arrows indicate damaged mitochondria; blue arrows indicate autophagosomes. Data in (A, B, H, I) are presented as mean ± SEM. For panels (A, B), two‐way ANOVA (treatment × time) with Tukey's post hoc test: Treatment *F* (1, 12), time *F* (2, 12), interaction *F* (2, 12). For survival curves (C, D): Log‐rank (Mantel‐Cox) test, df = 1 each. For motor function (F, G): Chi‐square test, df = 1 each. For panels (H, I): One‐way ANOVA with Tukey's post hoc test (four groups), *F* (3, 8). (E) Shows representative images without statistical comparison. *p < 0.05, **p < 0.01, ***p < 0.001.

To validate the role of AcSirt2 in aging in vivo, RNA interference (RNAi)‐mediated knockdown and pharmacological activation were performed in *A. cerana* . We synthesized dsRNA targeting AcSirt2 (Figure S5A,B) and delivered it to bees via oral feeding. RT‐qPCR and Western blotting results showed that compared with the *dsEGFP* control group, the mRNA and protein expression levels of AcSirt2 in bee brains were significantly suppressed at 24, 48, and 72 h post *dsAcSirt2* administration (Figure 5A,B), indicating effective silencing of AcSirt2 by RNAi. RT‐qPCR analysis further confirmed that the expression levels of other sirtuin family members (*AcSirt1*, *AcSirt4*–*AcSirt7*) were not significantly altered in bee brains at 72 h post *dsAcSirt2* treatment compared with the *dsEGFP* control (Figure S5C), ruling out off‐target effects. Furthermore, the transcriptional levels of two potential downstream genes involved in antioxidant defense, *AcSOD* and *AcCAT*, were also significantly decreased in the heads of the *dsAcSirt2* treated bees (Figure S5D,E).

Survival analysis revealed that *dsAcSirt2* treatment significantly shortened the median lifespan of worker bees, reducing the mean lifespan by 37.8% compared to the *dsEGFP* group (*dsEGFP*: 39.7 ± 2.8 days; *dsAcSirt2*: 24.7 ± 2.1 days; log‐rank test, df = 1, *χ* ^2^ = 34.2, *p* < 0.0001) (Figure 5C). For pharmacological treatments, the DMSO control group had a mean lifespan of 31.7 ± 1.2 days, which was shorter than the *dsEGFP* group (*p* < 0.01), reflecting the potential metabolic toxicity of long‐term 0.2% DMSO exposure. Notably, oral administration of the Sirt2 activator SRT1720 extended the mean lifespan by 42.9% compared to the DMSO control group (DMSO: 31.7 ± 1.2 days; SRT1720: 45.3 ± 5.5 days; log‐rank test, df = 1, *χ* ^2^ = 26.9, *p* < 0.0001) (Figure 5D).

Under identical starvation conditions, a significantly lower proportion of bees in the *dsAcSirt2* group rapidly reached the upper feeding platform (within 15 s) compared to the *dsEGFP* control (*χ* ^2^ test, df = 1, *p* < 0.01) (Figure 5F). Conversely, treatment with the Sirt2 activator SRT1720 significantly enhanced mobility, as evidenced by a higher success rate in reaching the platform than the DMSO control (*χ* ^2^ test, df = 1, *p* < 0.01; Figure 5G). To unequivocally confirm that the observed aging‐related phenotypes (e.g., shortened lifespan and impaired motor function) were due to AcSirt2 loss‐of‐function, we employed AK‐1, a specific Sirt2 inhibitor. AK‐1 treatment phenocopied the AcSirt2 knockdown, resulting in similarly shortened lifespan and impaired motor function (Figure S6A,B). Collectively, these results demonstrate that impaired AcSirt2 function is a primary cause of aging‐related phenotypic decline.

Brains of worker bees in the *dsAcSirt2* group exhibited obvious blue staining (a senescent characteristic), whereas those in the SRT1720‐treated group showed almost no blue staining, maintaining a youthful brain state (Figure 5E and Figure S7A,B). TEM ultrastructural analysis revealed that mitochondria in the brain cells of *dsAcSirt2*‐treated honey bees displayed varying degrees of swelling, vacuolization, cristae fragmentation, and even cristae loss, with a significant increase in the mitochondrial damage rate (*p* < 0.001) and a marked decrease in the number of autophagosomes in the cytoplasm (*p* < 0.01) (Figure 5H). In contrast, mitochondria in the brain cells of SRT1720‐treated honey bees maintained intact structures, and the number of autophagosomes was significantly increased (*p* < 0.01) (Figure 5I). Taken together, these findings indicate that AcSirt2 activity is closely associated with brain senescence and mitochondrial integrity in honey bees. Its inhibition promotes brain aging and mitochondrial damage while suppressing autophagy, whereas its activation preserves a youthful brain state and mitochondrial structure alongside enhanced autophagic activity.

### 2.6 AcSirt2 Interacts With FOXO and Mediates Its Deacetylation to Regulate Mitochondrial Function and Cellular Homeostasis

**FIGURE 6:**
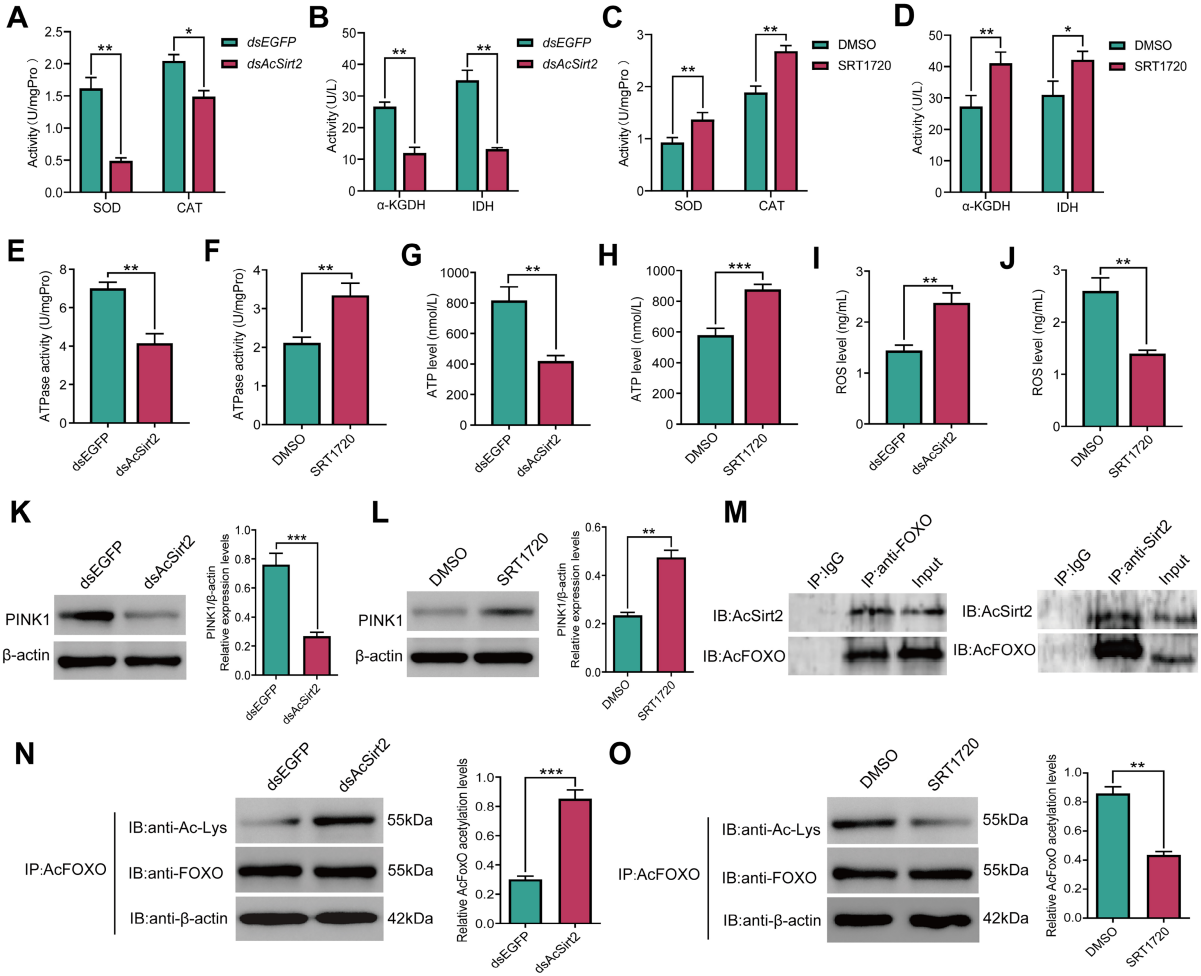
AcSirt2 interacts with FOXO and mediates its deacetylation to regulate mitochondrial function, autophagy, and enzyme activity. (A, B, E) Effects of AcSirt2 knockdown on the activities of antioxidant enzymes and metabolic enzymes in worker bee heads (*n* = 3). Enzyme activities include: SOD and CAT (A); α‐KGDH and IDH (B); ATPase (E). (C, D, F) Effects of AcSirt2 activation by SRT1720 on the activities of antioxidant enzymes and metabolic enzymes in worker bee heads (*n* = 3). Enzyme activities include: SOD and CAT (C); α‐KGDH and IDH (D); ATPase (F). (G, H) Effects of AcSirt2 knockdown (G) and SRT1720 treatment (H) on ATP levels in worker bee heads (*n* = 3). (I, J) Effects of AcSirt2 knockdown (I) and SRT1720 treatment (J) on ROS levels in worker bee heads (*n* = 3). (K, L) Protein levels of the mitophagy‐initiating protein PINK1 in worker bee heads upon AcSirt2 knockdown (K) or SRT1720 treatment (L) (*n* = 3). (M) Co‐IP assay confirming the in vivo interaction between AcSirt2 and AcFOXO (*n* = 3). (N, O) Relative acetylation level of AcFOXO (normalized to total AcFOXO) upon AcSirt2 knockdown (N) or SRT1720 treatment (O), as detected by Co‐IP/Western blot (*n* = 3). Data are presented as mean ± SEM. All comparisons were performed using unpaired Student's *t*‐test (df = 4 for each comparison). *p < 0.05, **p < 0.01, ***p < 0.001.

AcSirt2 knockdown significantly decreased the activities of antioxidant enzymes (SOD, CAT), key TCA cycle enzymes (α‐KGDH, IDH), and mitochondrial ATPase in the heads of worker bees (Figure 6A,B,E). In contrast, SRT1720 treatment markedly enhanced the activities of these enzymes (Figure 6C,D,F). These alterations in enzyme activities directly impacted cellular energy status and oxidative stress levels. Specifically, compared with the *dsEGFP* control, the *dsAcSirt2* group exhibited significantly decreased ATP levels (Figure 6G) and increased ROS levels (Figure 6I) in head tissues. SRT1720 treatment effectively elevated ATP content (Figure 6H) and reduced ROS accumulation (Figure 6J).

Western blot analysis further showed that AcSirt2 knockdown significantly reduced the expression of the mitophagy‐initiating protein PINK1 (Figure 6K), while SRT1720‐induced activation markedly increased PINK1 levels (Figure 6L). These molecular changes in PINK1 expression, consistent with the alterations in autophagosome number observed by TEM (Figure 5H,I), indicate that AcSirt2 participates in mitochondrial quality control by regulating mitophagy.

To explore the upstream regulatory mechanism, whether AcSirt2 interacted with the transcription factor AcFOXO was examined. Co‐immunoprecipitation (Co‐IP) assays confirmed a physical interaction between AcSirt2 and AcFOXO in vivo (Figure 6M). Crucially, the functional status of AcSirt2 directly determined the acetylation level of AcFOXO: AcSirt2 knockdown led to a significant increase in AcFOXO acetylation (Figure 6N), whereas SRT1720‐induced activation significantly decreased it (Figure 6O). The total FOXO protein levels in the immunoprecipitates remained consistent across groups (Figure 6N,O), confirming that the changes in acetylation level were specific post‐translational modifications.

Collectively, our results demonstrate that AcSirt2 interacts with AcFOXO and mediates its deacetylation. This functional interaction suggests that AcSirt2 may regulate downstream antioxidant, metabolic, and mitophagy networks through AcFOXO, thereby influencing aging‐related phenotypes.

## 3 Discussion

Employing the eastern honeybee *A. cerana* as a model system, we elucidated the molecular mechanism underlying AcSirt2‐mediated aging delay via the modulation of mitochondrial homeostasis. For the first time in a social insect, an AcSirt2‐FOXO‐mitophagy regulatory axis was established. Our data demonstrate that AcSirt2 physically interacts with the AcFOXO transcription factor, mediates its deacetylation, and thereby activates the PINK1/Parkin pathway to promote mitophagy, remodel the mitochondrial network, ameliorate cellular senescence, and ultimately delay aging in honey bees. This work not only refines the regulatory network of insect aging but also provides crucial evidence for the evolutionarily conserved function of the Sirtuin family, thereby establishing new experimental underpinnings for developing anti‐aging strategies.

Mitochondrial functional decline constitutes a core hallmark of aging across species, and its homeostatic maintenance relies on the precise coordination of mitochondrial dynamics and quality control mechanisms, including fusion, fission, and mitophagy (Lopez‐Otin et al. 2023; Lacombe and Scorrano 2024). Analysis of age‐related mitochondrial damage in the brains of *A. cerana* revealed a distinctive metabolic regulation pattern during aging in this social insect. YBs exhibited intact mitochondrial structures, whereas MBs displayed mitochondrial damage accompanied by a compensatory increase in autophagosome number, indicating early activation of autophagy to sustain metabolic homeostasis. However, this compensatory response failed in OBs, which showed severe mitochondrial structural damage and a decline in autophagic flux. The eventual reduction in autophagic efficiency leads to the accumulation of damaged mitochondria, thereby directly accelerating the aging process (Uoselis et al. 2023). This dynamic pattern aligns closely with the characteristics of mitochondrial quality decline observed in mammalian brain aging (Grimm and Eckert 2017; Sukhorukov et al. 2024) and is consistent with the biphasic regulation of autophagy reported in another aging model, *D. melanogaster* (Bai et al. 2012). These findings not only solidify the value of honey bees as a model for aging research but also provide new insights into the adaptive metabolic strategies of their short‐lived worker bees. Notably, AcSirt2 expression progressively decreases with age, suggesting its potential role as a key regulator of age‐associated mitochondrial functional decline. Functionally, AcSirt2 overexpression in both HaCaT and HEK‐293T cells not only alleviated H_2_O_2_‐induced senescence but also reduced the proportion of SA‐β‐gal‐positive cells, decreased ROS accumulation, and increased ATP levels, indicating conserved roles in oxidative stress resistance and energy metabolism regulation. Furthermore, AcSirt2 upregulates the expression of mitochondrial fusion and fission proteins to repair the fragmented mitochondrial network. This finding echoes the mechanism by which the Sir2 homolog regulates mitochondrial dynamics in yeast (Wang et al. 2019), further underscoring the high conservation of Sirtuin‐mediated mitochondrial network regulation across eukaryotes.

As a central pathway for clearing damaged mitochondria, mitophagy dysfunction is a key driver of accelerated aging (Diot et al. 2016; Chen et al. 2020; Picca et al. 2023). This study demonstrates that AcSirt2 maintains mitochondrial quality by coordinately regulating mitochondrial dynamics and mitophagy. Mechanistically, AcSirt2 overexpression reversed oxidative stress‐induced mitochondrial network fragmentation by upregulating the fusion proteins Mfn1 and Mfn2 and modulating the fission protein Drp1, thereby restoring damaged mitochondrial structure and maintaining network integrity. The coordinated interplay between mitochondrial dynamics and autophagy is known to be crucial for preserving mitochondrial quality (Twig and Shirihai 2011; Okamoto and Kondo‐Okamoto 2012; Li et al. 2025), suggesting that AcSirt2 may act as a key molecular switch in this regulatory coordination. Beyond mitochondrial dynamics, AcSirt2 also directly activates the PINK1/Parkin‐mediated mitophagy pathway, a conserved mechanism for selective degradation of damaged mitochondria (Narendra and Youle 2024). Recent studies reveal that PINK1 and Parkin not only mediate mitophagy but also redundantly regulate mitochondrial fusion through the metalloprotease OMA1 in mammals (Yamada et al. 2025). AcSirt2 overexpression significantly increased the LC3‐II/LC3‐I ratio and PINK1 protein levels and promoted Parkin recruitment to damaged mitochondria. These events are critical for initiating mitophagy, which involves the engulfment of mitochondria by autophagosomes and their subsequent degradation (Narendra and Youle 2024; Thayer et al. 2025). Critically, the mitophagy inhibitor 3‐MA substantially blocked the AcSirt2‐induced restoration of cellular antioxidant enzyme activities and ATP synthesis capacity, providing strong evidence that its cytoprotective effects are strictly dependent on a functional mitophagy pathway. Notably, simultaneous loss of PINK1‐Parkin in mice leads to mitochondrial over‐fusion, megamitochondria formation, and cGAS‐STING‐dependent inflammation, indicating that fusion inhibition is a conserved function of PINK1‐Parkin from *Drosophila* to mammals (Jiang 2025). By demonstrating that AcSirt2 activates the PINK1/Parkin‐mediated mitophagy pathway, this study establishes its role within the conserved framework of Sirt2‐mediated autophagy regulation, highlighting the potential of targeting AcSirt2 for therapeutic interventions in age‐related diseases.

Multi‐species studies support these findings. In mammals, SIRT2 enhances autophagic flux by upregulating the LC3‐II/LC3‐I ratio (Fang et al. 2024) and maintains cardiomyocyte homeostasis in primates by regulating STAT3 deacetylation, with its loss of function leading to accumulated mitochondrial damage and accelerated aging (Ye et al. 2023); in *D. melanogaster* , although Sirt2 has not been directly linked to the PINK1/Parkin pathway, it influences mitochondrial fragmentation by regulating the activity of the fission protein Drp1, indirectly providing substrates for mitophagy (Rana et al. 2017). Collectively, this evidence indicates that Sirt2 family proteins play a central and evolutionarily conserved role in regulating autophagy through diverse molecular mechanisms. The physiological relevance of this mechanism was confirmed in vivo. AcSirt2 knockdown shortened honey bee lifespan and increased mitochondrial damage rates in the brain, whereas SRT1720‐mediated activation of AcSirt2 effectively extended lifespan and preserved mitochondrial structural integrity. The consistency between these in vivo results and the in vitro cellular findings collectively confirms the physiological necessity of AcSirt2 in regulating healthy aging in insects.

The transcription factor FOXO is a core target of the Sirtuin family in aging regulation, with its activity precisely modulated by acetylation/deacetylation modifications (Park et al. 2015; Song et al. 2024). In this study Co‐IP assays confirmed a physical interaction between AcSirt2 and AcFOXO in *A. cerana* . Furthermore, assessment of acetylation levels demonstrated that AcSirt2 serves as a critical upstream deacetylase for AcFOXO, as AcSirt2 knockdown significantly increased AcFOXO acetylation, while its activation with SRT1720 markedly reduced it. This finding represents the first demonstration of a functional association between Sirt2 and FOXO in a social insect model, revealing an AcSirt2‐FOXO axis with remarkable evolutionary conservation. In mammals, SIRT2 regulates metabolic homeostasis and stress responses through FOXO deacetylation (Jing et al. 2007; Gomes et al. 2015; Huang et al. 2024); In *Caenorhabditis elegans* , SIR‐2.1 modulates the FOXO homolog DAF‐16 to improve mitochondrial homeostasis and extend lifespan (Mouchiroud et al. 2013; Zhang and Wang 2025). Notably, a study in *D. melanogaster* demonstrated that FOXO is essential for mediating the mitochondrial protective signal of Sir2 in PINK1 null mutants by showing that deletion of FOXO nullifies Sir2‐induced mitochondrial restoration, and that overexpression of FOXO or its downstream targets (e.g., SOD2) ameliorates PINK1 loss‐of‐function defects (Koh et al. 2012). Strikingly, this conserved Sirt2‐FOXO‐mitochondrial quality control axis has direct parallels in human neurodegenerative diseases. For instance, in Alzheimer's disease models, SIRT2 overexpression has been shown to attenuate mitochondrial dysfunction and neuronal toxicity, partly through modulating tau acetylation and autophagy (Esteves et al. 2019; Sola‐Sevilla et al. 2023). Conversely, FOXO3a, the mammalian ortholog, is recognized as a key neuroprotective factor whose activity is regulated by SIRT1/2‐mediated deacetylation, and its dysfunction is linked to increased neuronal vulnerability and impaired clearance of damaged organelles via mitophagy (Mouchiroud et al. 2013; Sun et al. 2018). Integrated with our transcriptomic data, we propose a model whereby AcSirt2‐mediated deacetylation of AcFOXO transcriptionally activates downstream target genes such as *LC3*, *PINK1*, and *SOD*, thereby coordinately enhancing mitophagy, antioxidant defense, and energy metabolism to delay aging. Notably, although our results confirm the upstream regulatory relationship of the AcSirt2‐AcFOXO axis on the PINK1/Parkin mitophagy pathway, the direct transcriptional activation of PINK1 by AcFOXO has not been conclusively verified in the present study. We propose this regulatory cascade as a reasonable mechanistic hypothesis based on robust mammalian studies, which have demonstrated that mammalian FOXO3a can directly bind the PINK1 promoter and initiate PINK1 transcription to modulate mitophagy (Mei et al. 2009; Sengupta et al. 2011; Gupta et al. 2022). Further specific molecular verification is still required to confirm whether AcFOXO directly targets the *PINK1* gene or regulates PINK1 expression through indirect intermediate mechanisms in honeybees.

In addition, a study in *D. melanogaster* (Koh et al. 2012) also provides insights into the pathway hierarchy and species‐specific adaptations. In their genetic interaction model, Sir2 and FOXO function downstream of PINK1, as PINK1 deletion produces no additive deleterious effect on dopaminergic neuron loss in Sir2 or FOXO mutants, supporting a common protective pathway for PINK1, Sir2, and FOXO (Koh et al. 2012; Han et al. 2021). This is consistent with our finding that AcSirt2 activates PINK1 expression, suggesting a conserved regulatory axis where Sirt2‐FOXO acts in concert with PINK1. An interesting divergence, however, concerns the requirement for Parkin. While Koh et al. (2012) reported that *D. melanogaster* Sir2 acts independently of Parkin, our study demonstrates that AcSirt2 activates the PINK1/Parkin pathway. This speciesspecific difference in Parkin engagement may reflect evolutionary divergence i‐n mitophagy regulation between social and solitary insects. Specifically, as a social insect with a highly organized caste system and division of labor, *A. cerana* may have evolved more stringent mitochondrial quality control mechanisms to sustain the prolonged metabolic demands of worker bees and ensure colony fitness. The recruitment of Parkin‐mediated mitophagy, which provides robust clearance of damaged mitochondria through ubiquitin‐dependent signaling, could represent an evolutionary adaptation to enhance cellular homeostasis under the high‐energy conditions associated with social living. Consistent with this hypothesis, studies in *Drosophila* and mammalian tissues have shown that basal mitophagy can proceed through PINK1/Parkin‐independent receptor‐mediated pathways (e.g., BNIP3, NIX), particularly under conditions where rapid mitochondrial turnover is required (Mei et al. 2009). In contrast, the solitary life history of *D. melanogaster* , with its shorter lifespan and less complex social structure, may tolerate less elaborate mitochondrial surveillance systems, rendering Parkin dispensable for Sir2‐mediated neuroprotection (Koh et al. 2012). This divergence is further supported by the observation that Parkin‐independent mitophagy pathways are more prominently utilized in *Drosophila* and certain mammalian tissues, whereas the PINK1/Parkin axis appears more dominant in long‐lived or metabolically active organisms (Steffan et al. 2025). Alternatively, this discrepancy may also stem from tissue‐specific or context‐dependent differences in mitophagy regulation, as Koh et al. (2012) focused on dopaminergic neurons, whereas our study examined brain tissue broadly. Therefore, whether the Parkin‐dependent versus Parkin‐independent modes reflect genuine evolutionary divergence between social and solitary insects, or simply distinct tissue‐specific requirements, warrants systematic comparative investigation across multiple hymenopteran and dipteran species. Elucidating the evolutionary dynamics of Sirt2‐FOXO‐PINK1/Parkin modules will not only refine our understanding of insect aging diversity but also provide insights into how sociality shapes mitochondrial quality control strategies.

An important observation in this study is the significant lifespan shortening and mitochondrial damage induced by chronic oral administration of low‐concentration (0.2%) DMSO, which served as the vehicle control. This aligns with growing evidence that DMSO, even at concentrations traditionally considered safe for acute exposure, can exert toxicity upon prolonged contact in various models. In honeybees, DMSO has been implicated in reduced sperm viability leading to queen sterility, impaired brood survival likely via effects on egg quality or nurse bee care, and induction of colony stress responses such as absconding behavior (Wegener and Bienefeld 2012; Milchreit et al. 2016). More broadly, studies in zebrafish embryos and mammalian neural cells have shown that DMSO can induce developmental delays, compromise mitochondrial integrity, and disrupt membrane potential, leading to mitochondrial swelling and increased generation of reactive oxygen species (Galvao et al. 2014; Yuan et al. 2014; Kim and Lee 2021). Our observation of mitochondrial ultrastructural damage and elevated ROS in DMSO‐treated honeybee brains is consistent with this reported impact on mitochondrial integrity (Yuan et al. 2014). Therefore, the premature aging phenotype in our DMSO control group likely results from a combination of metabolic disturbance and chronic oxidative stress induced by the solvent. This underscores the critical need for careful solvent optimization and interpretation of data in long‐term invertebrate feeding studies. Importantly, the fact that SRT1720 treatment significantly extended lifespan and improved mitochondrial metrics relative to this DMSO‐stressed baseline strongly reinforces the potency of AcSirt2 activation in counteracting not only natural aging but also externally induced mitochondrial dysfunction and oxidative stress.

Despite systematically elucidating the anti‐aging mechanism of AcSirt2, several important research directions warrant further exploration in the future. First, while we demonstrated the conserved function of AcSirt2 in human cell lines, its genuine physiological role within honey bees requires further validation using primary cells or tissues. Second, although we confirmed that AcSirt2 exerts its effects through FOXO deacetylation, direct evidence illustrating how deacetylated FOXO transcriptionally activates its downstream target genes remains limited. Future investigations should employ genome‐wide techniques such as ChIP‐seq or CUT&Tag to map FOXO binding sites and validate specific targets (Bansal et al. 2015; Xiong et al. 2024). Third, although Co‐IP assays confirmed the physical interaction between AcSirt2 and AcFOXO in vivo, this approach cannot discriminate direct binding from indirect associations; therefore, further validation using methods such as GST pull‐down or yeast two‐hybrid is warranted to conclusively demonstrate a direct physical interaction. Fourth, the regulatory complexity of the AcSirt2‐FOXO axis itself warrants further exploration. It remains to be determined whether their interaction is mediated or cooperatively regulated by other co‐regulators, or is cross‐regulated by other post‐translational modifications such as phosphorylation and ubiquitination. Moreover, as this work primarily focused on the brain, the tissue‐specific functions of AcSirt2 in other key organs (Ma et al. 2024), such as the fat body and ovaries, warrant exploration and may uncover new dimensions of aging regulation. Finally, the SRT1720 compound used here is a pan‐Sirt2 activator with potential off‐target effects on other sirtuin family members (Dai et al. 2018). Thus, validating these phenotypes using more specific chemical probes or genetic tools is essential.

In summary, our study unveils a novel mechanism whereby AcSirt2 regulates mitochondrial homeostasis and autophagy via AcFOXO deacetylation. This work not only enriches the molecular network of insect aging but also identifies new candidate targets for anti‐aging research. Future efforts to delineate the downstream targetome of AcFOXO and to develop specific AcSirt2 modulators hold promise for extending honey bee lifespan, improving pollination efficiency, and providing cross‐species insights for combating human age‐related diseases.

## 4 Conclusion

This study systematically elucidated the critical role and molecular mechanism of *A. cerana* Sirt2 (AcSirt2) in delaying aging. We found that AcSirt2 expression decreases with age, and its loss of function exacerbates mitochondrial damage, impairs autophagic capacity, and shortens lifespan. Mechanistically, AcSirt2 interacts with the FOXO transcription factor and mediates its deacetylation, thereby activating the PINK1/Parkin‐mediated mitophagy pathway and promoting the expression of mitochondrial fusion/fission proteins (Mfn1, Mfn2, Drp1) to remodel the mitochondrial network. These changes collectively enhance cellular antioxidant defense, improve energy metabolic homeostasis, and ultimately delay the aging process. This study is the first to fully delineate an AcSirt2‐FOXO‐mitophagy regulatory axis in a social insect, extending the Sirtuin‐mediated aging regulatory mechanism from classic model organisms to honey bees with complex social behavior, thereby providing new experimental evidence for understanding the evolutionary principles of aging regulation networks. Future studies could further explore the tissue‐specific functions of AcSirt2 in organs such as the fat body and ovary, map the genomic binding landscape of FOXO using techniques such as ChIP‐seq, and generate specific mutants by constructing CRISPR/Cas9 gene‐edited bee lines, thereby establishing a more solid theoretical foundation for in‐depth analysis of aging mechanisms in social insects and the development of precise anti‐aging strategies.

## 5 Methods

### 5.1 Rearing of *Apis cerana* and Sample Collection

Worker bee samples of *A. cerana* used in this study were collected from the beekeeping base of Chongqing Normal University (29.36°N, 106.17°E). All experimental procedures adhered to internationally accepted ethical standards for invertebrate research, ensuring that sample collection and processing complied with animal welfare requirements (Elwood 2011; Pollo and Vitale 2019). To obtain worker bees of precise ages, we followed a previously established method (Ma et al. 2025): newly emerged worker bees within 24 h of eclosion were obtained through indoor constant‐temperature incubation, marked on the thorax, and then returned to their original colonies. Subsequently, the marked bees were recaptured and divided into three age groups based on their post‐eclosion days: young bees (YBs, 1~5 days post‐eclosion, dpe), mid‐aged bees (MBs, 29 dpe), and old bees (OBs, 50 dpe). Worker bees collected from different age groups were anesthetized on ice, their heads were rapidly separated, snap‐frozen in liquid nitrogen, and stored at −80°C for subsequent RNA extraction, protein isolation, and biochemical analyses.

### 5.2 Transmission Electron Microscopy (TEM)

Samples of bee brain tissue and cultured cells were prepared separately for TEM. Brains were dissected from the heads of bees of different ages on ice. Throughout the dissection, ice‐cold phosphate‐buffered saline (PBS, pH 7.4) was applied to keep the tissue moist and minimize degradation. Cell samples (HaCaT and HEK‐293T cells) were collected by centrifugation (110 × g, 5 min, 4°C) and washed twice with PBS to remove residual culture medium. All samples were fixed overnight at 4°C with 2.5% glutaraldehyde. Subsequent common processing steps were identical: samples were washed three times with 0.1 M PBS, post‐fixed in 1% osmium tetroxide for 2 h, then dehydrated through a graded acetone series (30%, 50%, 70%, 80%, 90%, 95%, and 100%), with each step lasting 15 min. Following dehydration, the samples were embedded in Epon 812 resin and polymerized at 60°C for 48 h to complete the embedding process. Ultrathin sections (60 nm thickness) were cut using an ultramicrotome (Leica UC7rt, Germany) and mounted on copper grids. The sections were stained with 2% uranyl acetate for 10~15 min at room temperature, followed by staining with lead citrate for 1~2 min. Finally, the stained sections were observed under a transmission electron microscope (JEOL JEM‐1400FLASH, Japan) to examine ultrastructural features, including mitochondrial damage and autophagosome count. Image J 1.50 software (NIH, USA) was used to analyze and quantify the number of autophagosomes and mitochondrial morphology.

### 5.3 Detection of Tissue ATP and ROS Levels

To measure ATP and ROS levels in the brain tissue of worker bees under different ages or treatments, corresponding enzyme‐linked immunosorbent assay (ELISA) kits were used. Tissue samples were homogenized mechanically in an ice bath with pre‐cooled PBS (pH 7.4) at a mass‐to‐volume ratio of 1:9. The homogenates were then centrifuged at 860 × g for 20 min at 4°C, and the resulting supernatants were carefully collected for analysis. ATP content was measured using an Insect ATP ELISA Kit (Cat. No. EHJ‐99905k, HuiJia Biotechnology, China). ROS levels were determined with an Insect ROS ELISA Kit (Cat. No. EHJ‐98163, HuiJia Biotechnology, China). All procedures were performed in strict accordance with the manufacturers' instructions. Briefly, after setting up standard and sample wells and adding samples and reagents, plates were incubated at 37°C as per the kit protocols. This was followed by washing, color development, and reaction termination steps. The absorbance of each well was immediately measured at 450 nm using a microplate reader (SpectraMax 190, USA). The concentrations of ATP and ROS in the samples were calculated based on standard curves generated on the same day. Final data were expressed as content per gram of tissue (nmol/L or ng/mL). Each experiment included no fewer than three biological replicates.

### 5.4 Tissue Immunofluorescence Assay

To detect the expression and localization of LC3B in the brains of worker bees at different ages, brains from YB, MB, and OB groups were collected separately, dissected, fixed with 4% paraformaldehyde, embedded in paraffin, and sectioned into 5 μm‐thick slices. After dewaxing, rehydration, and heat‐mediated antigen retrieval with sodium citrate buffer (pH 6.0), the sections were blocked with 5% bovine serum albumin (BSA) at room temperature for 1 h. Subsequently, the sections were incubated with rabbit anti‐LC3B monoclonal antibody (Cat. No. ab192890, Abcam, USA) (1:200 dilution) at 4°C overnight in the dark. On the next day, after washing with PBS, the sections were incubated with FITC‐conjugated goat anti‐rabbit IgG (1:500 dilution) in the dark at room temperature for 1 h, followed by nuclear counterstaining with DAPI. Finally, the sections were mounted with antifade mounting medium, and images were captured under a fluorescence microscope (Olympus, Germany). The expression changes of the target protein in the brains of worker bees from different age groups were evaluated by analyzing fluorescence intensity using ImageJ 1.50 software.

### 5.5 Transcriptomic Data Analysis

This study utilized our previously published transcriptomic dataset (Ma et al. 2025), which contained Illumina sequencing data derived from head of *A. cerana* worker bees across different age groups (YB, MB, and OB). The raw data are publicly available in the NCBI Sequence Read Archive under the accession number PRJNA1234368. Differential expression analysis was performed using DESeq2 software (version 1.38.3). From this analysis, *Sirt2* was identified as a candidate gene associated with aging and mitochondrial function for subsequent experimental validation.

### 5.6 Plasmid Construction

The *A. cerana* *Sirt2* (*AcSirt2*, NCBI Gene ID: LOC107993112) has three annotated mRNA variants (XM_062080102.1, XM_017049343.2, and XM_017049342.3). The present study utilized the longest transcript, XM_062080102.1, which encodes the full‐length AcSirt2 protein containing the conserved NAD^+^‐binding pocket and catalytic deacetylase domain, for all functional experiments. This coding sequence (NCBI RefSeq: XM_062080102.1) was cloned into the pcDNA3.1(+) vector via *HindIII* and *XhoI* sites using primers listed in Table S2 to generate the *AcSirt2* overexpression plasmid, pcDNA3.1‐*AcSirt2*. The resulting construct includes in‐frame fusions with GFP and a 3 × Flag tag for protein detection and visualization.

### 5.7 Cell Culture and Transfection

The human immortalized keratinocyte (HaCaT) and human embryonic kidney (HEK‐293T) cell lines used in this study were routinely maintained in our laboratory. Both cell lines were cultured in high‐glucose DMEM supplemented with 10% fetal bovine serum and 1% penicillin–streptomycin at 37°C in a humidified incubator with 5% CO_2_. For experiments, cells were seeded in 24‐well plates at a density of 5.0 × 10^5^ cells per well and cultured for 24 h until they reached approximately 70~80% confluence. Transfection was then performed using Lipofectamine3000 (Invitrogen, USA) according to the manufacturer's instructions, introducing either the empty pcDNA3.1 vector or the recombinant pcDNA3.1‐*AcSirt2* plasmid. At 48 h post‐transfection, transfection efficiency was initially assessed by direct observation of GFP fluorescence using a fluorescence microscope. Cells were then harvested for total protein extraction, and AcSirt2 protein expression was further verified by Western blotting using an anti‐Flag antibody.

### 5.8 Establishment and Validation of the Cellular Senescence Model

To establish an oxidative stress‐induced cellular senescence model, H_2_O_2_ was chosen as the senescence inducer because it is a stable and membrane‐permeable reactive oxygen species that reliably induces oxidative stress, causes mitochondrial dysfunction, and triggers stress‐induced premature senescence (SIPS). H_2_O_2_ is the most widely used and best‐characterized inducer for in vitro cellular senescence models (Wang et al. 2013; Wei et al. 2025). HaCaT and HEK‐293T cells in the logarithmic growth phase were seeded in 96‐well plates at a density of 1 × 10^5^ cells/mL. After 24 h of adhesion, the medium was replaced with fresh medium containing a range of H_2_O_2_ concentrations (0, 50, 100, 200, 300, 400, 600, and 800 μM), followed by further incubation for 12 h or 24 h to screen for optimal senescence induction conditions. Cell viability was assessed using the Cell Counting Kit‐8 (CCK‐8) assay (see below for detailed protocol). Dose‐dependent decrease in viability were observed in both cell lines (Figure S8A–D). Treatment with 200 μM H_2_O_2_ for 24 h reduced HaCaT cell viability to 63.2%, whereas 300 μM H_2_O_2_ for 24 h reduced HEK‐293T cell viability to 64.1%. Senescence‐associated β‐galactosidase (SA‐β‐gal) staining (see below for detailed protocol) further confirmed a significant increase in senescent cells under these conditions (Figure S8E–H). Based on these data, a 24‐h treatment with 200 μM or 300 μM H_2_O_2_ was selected as the standard condition for inducing senescence in HaCaT and HEK‐293T cells, respectively, in all subsequent experiments. Once the induction conditions were determined, the following experimental groups were established: a normal control group (untreated), an H_2_O_2_ model group (treated only with H_2_O_2_), an empty vector control group (H_2_O_2_ + Vector, transfected with pcDNA3.1 empty plasmid after H_2_O_2_ treatment), and an AcSirt2 overexpression group (H_2_O_2_ + *AcSirt2*, transfected with recombinant pcDNA3.1‐*AcSirt2* plasmid after H_2_O_2_ treatment). To investigate the role of AcSirt2 in mitophagy, an additional intervention group was included in which cells were treated with 5 mM of the mitophagy inhibitor 3‐methyladenine (3‐MA) following transfection with pcDNA3.1‐*AcSirt2*.

### 5.9 Cell Viability Assay

Cell viability was measured using a Cell Counting Kit‐8 (CCK‐8, MCE, Cat. No. HY‐K0301, USA) according to the manufacturer's instructions. Briefly, after completing the respective treatments, 10 μL CCK‐8 solution was added to each well of a 96‐well plate. The plate was then incubated at 37°C in a humidified atmosphere of 5% CO_2_ for 2 h in the dark. Subsequently, optical density (OD) at 450 nm was measured for each well using a microplate reader (SpectraMax 190, USA). The CCK‐8 assay exhibits a well‐established linear correlation between the OD450 nm signal and the number of viable cells within the range used in our experiments; therefore, cell viability was expressed as a percentage relative to the normal control group (set as 100%), calculated using the formula: Cell viability (%) = (OD_treated_/OD_normal_) × 100%. Each experimental group contained five replicate wells, and all experiments were independently repeated three times.

### 5.10 Senescence‐Associated β‐Galactosidase (SA‐β‐Gal) Staining

After completion of the respective treatments, the target cells were rinsed with PBS and fixed with 0.2% glutaraldehyde for 15 min at room temperature. Fresh SA‐β‐gal staining solution was then prepared in strict accordance with the manufacturer's instructions provided with the SA‐β‐gal Staining Kit (Beyotime, Cat. No. CP602, China). The cells were incubated in this staining solution at 37°C in a CO_2_‐free incubator for 4~12 h. Following incubation, multiple random fields of view (≥ 3) were observed and imaged using a light microscope (Nikon Eclipse Ts2, Japan). The percentage of SA‐β‐gal‐positive cells (stained blue) was calculated, with a minimum of 500 cells counted per experimental group to ensure reliable quantification.

### 5.11 Determination of Intracellular Reactive Oxygen Species (ROS) Levels

Intracellular ROS levels were evaluated using the fluorescent probe CM‐H2DCFDA (Cat. No. S0035S, Beyotime, China). Target cells were incubated with 10 μM CM‐H2DCFDA at 37°C in the dark for 30 min. After incubation, the cells were washed three times with PBS to remove unbound probe. Images were observed and captured using an inverted fluorescence microscope (Nikon Eclipse Ti2, Japan). Fluorescence intensity was measured at an excitation wavelength of 495 nm and an emission wavelength of 530 nm using a multimode microplate reader (Varioskan LUX, Thermo Fisher, USA). Each experiment included three biological replicates. Relative ROS levels in each group were calculated with reference to the fluorescence intensity of the control group.

### 5.12 Measurement of Intracellular ATP Levels

Intracellular ATP levels were measured using the CellTiter‐Glo Luminescent Assay Kit (Cat. No. G7570, Promega, USA) according to the manufacturer's protocol. Briefly, cells were seeded in white 96‐well plates at a density of 1 × 10^4^ cells per well, cultured overnight to allow adherence, and subjected to the respective treatments. After discarding the original culture medium, an equal volume of CellTiter‐Glo Reagent was added to each well. The plate was shaken on an orbital shaker in the dark for 2 min to ensure complete cell lysis, followed by incubation at room temperature for 10 min to stabilize the luminescent signal. Finally, the luminescence intensity of each well was measured using a multimode microplate reader (Varioskan LUX, Thermo Scientific, USA). The ATP content in the samples was calculated by comparing the measured values against a standard curve constructed with ATP of known concentrations.

### 5.13 Mito‐Tracker Red Fluorescent Staining

Mitochondrial mass in HaCaT and HEK‐293T cells was evaluated using the fluorescent probe Mito‐Tracker Red CMXRos (Cat. No. C1035, Beyotime, China). Following the manufacturer's instructions, a fresh working solution was prepared at a final concentration of 200 nM. Cells subjected to the designated treatments were incubated with this working solution at 37°C in the dark for 30 min. After incubation, the working solution was removed, and fresh cell culture medium was added. Observations and image acquisition were performed using an inverted fluorescence microscope (Nikon Eclipse Ti2, Japan). Quantitative analysis of mitochondrial fluorescence intensity was conducted using Image J 1.50 software on at least five randomly selected fields of view.

### 5.14 Enzyme Activity Assays

*A. cerana* brains and treated HaCaT/HEK‐293T cells were collected separately to prepare tissue homogenates or cell lysates. The protein concentration of each sample was determined using the BCA method for normalization. Subsequently, the activities of the following enzymes were measured strictly according to the manufacturers' instructions of the respective commercial kits: total superoxide dismutase (SOD, Cat. No. S0101S, Beyotime, China), catalase (CAT, Cat. No. S0051, Beyotime, China), isocitrate dehydrogenase (IDH, Cat. No. S0526S, Beyotime, China), ATPase (Cat. No. A070‐1‐2, Jiancheng Bioengineering Institute, China), and α‐ketoglutarate dehydrogenase (α‐KGDH, Cat. No. KGDH‐1‐Y, Keming Biotechnology, China). Briefly, samples were mixed with reaction reagents at the ratio specified in the kit instructions and incubated at the designated temperature until the reaction reached completion. The absorbance values of each sample were measured at the wavelength corresponding to each enzyme using a microplate reader. Finally, the specific activity of each enzyme (U/mg prot) was calculated based on the standard curve generated for each enzyme. All experiments were performed with three independent biological replicates.

### 5.15 Quantitative Real‐Time PCR (RT‐qPCR)

Honey bee brains and treated HaCaT/HEK‐293T cells were collected separately. Tissue samples were snap‐frozen in liquid nitrogen and homogenized using a cryogenic grinder with TRIzol reagent (Invitrogen, USA), while cell samples were directly lysed with TRIzol. Total RNA was then extracted via standard procedures including chloroform extraction, isopropanol precipitation, and 75% ethanol washing, and the RNA pellet was finally dissolved in DEPC‐treated water. The concentration and purity of RNA were determined using a NanoDrop One spectrophotometer (ThermoFisher, USA). For cDNA synthesis, 1 μg of total RNA was reverse‐transcribed following the instructions of the GoScript Reverse Transcription System (Promega, USA). Quantitative PCR amplification was performed on a CFX96 Touch Real‐Time PCR Detection System (Bio‐Rad, USA) using SYBR Green Master Mix (Novogene, China). The reaction protocol was as follows: initial denaturation at 95°C for 30 s, followed by 39 cycles of denaturation at 95°C for 15 s and annealing/extension at 60°C for 10 s. After amplification, a melting curve analysis was performed to confirm the specificity of the PCR products. β‐actin from *A. cerana* or *Homo sapiens* was used as the internal reference gene, respectively, and the relative expression levels of target genes were calculated using the 2^−ΔΔCt^ method. The sequences of the RT‐qPCR primers used in this study are listed in Table S2.

### 5.16 RNA Interference and SRT1720 Treatment in *A. cerana*

Double‐stranded RNAs (dsRNAs) targeting *AcSirt2* (*dsAcSirt2*) and a non‐targeting control dsRNA (*dsEGFP*) were synthesized in vitro using the T7 RiboMAX Express RNAi System (Promega, USA). The primer sequences used for dsRNA synthesis are listed in Table S2. The specificity of *dsAcSirt2* primers was verified by NCBI Primer‐BLAST to ensure exclusive targeting of AcSirt2 without cross‐reactivity to other sirtuin family members (*AcSirt1*, *AcSirt4*–*AcSirt7*). To further rule out off‐target effects, the expression levels of non‐target sirtuin family members (*AcSirt1*, *AcSirt4*–*AcSirt7*) were examined by RT‐qPCR in bee heads at 72 h post‐treatment using primers validated previously (Ma et al. 2024). Newly emerged honey bees were randomly divided into the following groups and fed the following diets: (1) the *dsEGFP* group, receiving sucrose syrup supplemented with *dsEGFP* (2 μg/μL); (2) the *dsAcSirt2* group, receiving sucrose syrup supplemented with *dsAcSirt2* (2 μg/μL).

To pharmacologically modulate Sirt2 activity, we prepared stock solutions of the activator SRT1720 (MCE, USA) and the inhibitor AK‐1 (MCE, USA) in dimethyl sulfoxide (DMSO, ≥ 99.9% cell culture grade, Sigma‐Aldrich, USA) at a concentration of 5 mM. Prior to the formal experiment, a solvent toxicity assessment was conducted to determine a tolerable concentration of DMSO for chronic oral administration. Newly emerged worker bees were randomly divided into six groups (50 bees per group, *n* = 3 biological replicates). Five DMSO‐treated groups fed with 50% (w/v) sucrose syrup containing DMSO at final concentrations of 0.2%, 0.4%, 0.6%, 0.8%, or 1.0% (v/v), and one age‐matched control group fed with pure 50% (w/v) sucrose syrup (without DMSO). All groups were reared under the same environmental conditions to eliminate batch effects. Survival was monitored daily. Bees exposed to 0.6~1.0% DMSO died within 10 days, and the 0.4% DMSO group had a median lifespan of only 12 days. In contrast, the 0.2% DMSO group exhibited a median lifespan of approximately 24 days (Figure S9). Although this was shorter than the median lifespan of untreated bees in our laboratory conditions (~27 days), it represented the least adverse effect among all DMSO concentrations tested and provided a sufficient observation window for evaluating Sirt2 modulation. Therefore, 0.2% DMSO was selected as the solvent control for all subsequent long‐term pharmacological experiments.

For both the SRT1720 and AK‐1 treatment groups, a 5 mM stock solution of each compound was diluted in 50% sucrose syrup to a final concentration of 10 μM compound and 0.2% DMSO. Specifically, 10 μL of the respective 5 mM stock was added to 5 mL of syrup. Corresponding control groups for each compound received syrup containing an equal volume of DMSO (10 μL in 5 mL syrup; final concentration 0.2% DMSO) without the compound. The syrup was replaced daily, and survival rates were recorded every day to generate survival curves.

### 5.17 Whole‐Mount Staining of SA‐β‐Gal Activity in *A. cerana* Brains

Whole‐mount senescence‐associated β‐galactosidase (SA‐β‐gal) staining of *A. cerana* worker bee brains was performed using a protocol adapted from (Byrns et al. 2024). Briefly, whole brains from worker bees of different treatment groups were rapidly dissected in ice‐cold PBS and fixed for 40 min at room temperature in fixation solution containing 2% paraformaldehyde and 0.2% glutaraldehyde. After fixation, tissues were washed three times with PBS (5 min per wash). Brains were then incubated in 200 μL of commercial SA‐β‐gal staining solution (Cat. No. CP602, Beyotime, China) at 37°C for 12 h in the dark with continuous shaking (300 rpm). Post‐staining, tissues were washed three times with PBS (5 min each). To enable clear optical observation, tissues were cleared overnight in an anti‐fade mounting medium (20 mM Tris pH 8.0, 0.5% N‐propyl gallate, 80% glycerol in PBS). Images of the whole brains were acquired using a stereomicroscope (Leica S9E, Germany).

### 5.18 Locomotor Activity Assay

Locomotor activity of *A. cerana* worker bees was assessed using a cup‐based apparatus, as originally described by Peleg et al. (2016), following the specific protocol from our previous study (Ma et al. 2025). Briefly, the custom‐made device consists of two transparent cups. A sponge ball, soaked in 50% sucrose solution and serving as a feeding platform, was fixed at the bottom of the upper cup. Worker bees from different treatment groups (40 bees per group; *n* = 3 biological replicates) were starved for 2 h under standard conditions and then placed into the lower chamber. After removal of the separating plastic film between the two chambers, the number of bees that successfully climbed to the upper cup platform within 15 s was recorded. Immediately after counting, the plastic separator was reinserted to secure the bees on the platform. The success rate was calculated and used as a measure of locomotor performance.

### 5.19 Western Blot

Total protein was extracted from *A. cerana* worker bee brains as well as treated HaCaT and HEK‐293T cells. Tissue samples were flash‐frozen in liquid nitrogen and thoroughly homogenized in pre‐cooled RIPA lysis buffer supplemented with 1% PMSF using a cryogenic grinder (JXFSTPRP‐CLN, Jingxin, China). Cell samples were harvested with a cell scraper, washed with PBS, and lysed in RIPA lysis buffer on ice. All lysates were centrifuged at 13680 × g for 15 min at 4°C, and the supernatants were collected as total protein. Protein concentration was determined using the BCA method. A total of 60 μg protein per sample was mixed with 5× SDS‐PAGE loading buffer, denatured by heating at 100°C for 15 min, and separated by SDS‐PAGE. The proteins were then transferred onto a PVDF membrane (Millipore, Germany). The membrane was blocked with 5% skim milk for 2 h at room temperature, followed by incubation with appropriate primary antibodies at 4°C overnight. After washing with TBST, the membrane was incubated with HRP‐conjugated secondary antibodies for 1 h at room temperature. Protein bands were visualized using an ECL detection system (JS‐M6, Shanghai, China), and band intensities were quantified with Image J 1.50 software using β‐actin as the loading control.

### 5.20 Co‐Immunoprecipitation (Co‐IP)

To verify the interactions between AcSirt2 and AcFOXO proteins, co‐immunoprecipitation (Co‐IP) was performed. Protein A + G magnetic beads (Cat. No. P2055, Beyotime, China) were separately conjugated with 2 μg mouse anti‐AcSirt2 or rabbit anti‐FOXO polyclonal antibody (in‐house generated) by overnight incubation at 4°C. Corresponding species‐matched IgG was used as a negative control. After antibody conjugation, freshly prepared total protein extracts from *A. cerana* worker bee heads (adjusted to a concentration of 500 μg/mL) were added to each bead‐antibody complex, followed by 6‐h co‐incubation at 4°C with gentle rotation. The complexes were thoroughly washed with Western and IP cell lysis buffer (Cat. No. P0013, Beyotime, China), and the bound proteins were eluted by boiling with 1× SDS loading buffer. Finally, cross‐validation was performed by Western blotting: precipitates from the anti‐AcSirt2 antibody were detected with the anti‐FOXO antibody, and precipitates from the anti‐FOXO antibody were detected with the anti‐AcSirt2 antibody. An Input control was included in the assay.

### 5.21 Detection of FOXO Acetylation Level

The acetylation level of FOXO was detected by Co‐IP combined with Western blotting. Briefly, total protein was extracted from different treated *A. cerana* worker bee heads using RIPA lysis buffer supplemented with deacetylase inhibitors (10 mM nicotinamide and 1 μM trichostatin A). For each Co‐IP reaction, 500 μg total protein was incubated overnight at 4°C with an anti‐FOXO antibody, followed by precipitation with Protein A/G magnetic beads. After extensive washing with Western and IP cell lysis buffer, the immunoprecipitated complexes were subjected to Western blotting. The membrane was first probed with an anti‐acetylated lysine antibody (Cat. No. PTM‐105RM, PTMBIO, China) to detect acetylated FOXO levels. Subsequently, the same membrane was stripped and reprobed with an anti‐FOXO antibody to determine the total amount of immunoprecipitated FOXO, which served as the loading control. The relative acetylation level was quantified by normalizing the intensity of the acetylated FOXO band to that of the total FOXO band.

### 5.22 Statistical Analyses

Data were presented as the mean ± standard error of the mean (SEM). Statistical analyses were performed using GraphPad Prism 6.02 software (GraphPad Inc., USA). Differences between two groups were analyzed by unpaired Student's *t*‐test (df = *n*₁ + *n*_2_–2). Multiple groups were compared using one‐way or two‐way analysis of variance (ANOVA) followed by Tukey's post hoc test, with *F* (DFn, DFd) reported accordingly (for one‐way ANOVA: DFn = *k*−1, DFd = *N*−*k*, where *k* is the number of groups and *N* is the total sample size; for two‐way ANOVA, degrees of freedom for individual factors and their interaction are reported separately). The Chi‐square (*χ* ^2^) test (df = 1 for 2 × 2 contingency tables) was used for proportion comparisons, and the Log‐rank (Mantel–Cox) test (df = number of groups −1) was applied for survival analysis. A *p*‐value of less than 0.05 was considered statistically significant. Exact sample sizes and degrees of freedom for each test are specified in the figure legends.

## Author Contributions

**Qiang Ma:** conceptualization, methodology, formal analysis, data curation, writing – original draft, visualization. **Zhengang Ma:** validation, resources, writing‐original draft, writing – review and editing, funding acquisition, project administration. **Tingyue Huang:** validation, data curation, investigation. **Qianmin Hai:** methodology, validation, data curation. **Xiaoqun Dang:** methodology, writing – review and editing, funding acquisition. **Jinshan Xu:** visualization, software, funding acquisition. **Jialing Bao:** methodology, formal analysis, writing – review and editing. **Zachary Y. Huang:** writing – review and editing, formal analysis, methodology. **Zeyang Zhou:** resources, writing – review and editing, supervision, funding acquisition. All authors have read and approved the final manuscript.

## Funding

This work was supported by the National Natural Science Foundation of China, 32372944, 31770160. The Natural Science Foundation of Chongqing, CSTB2025NSCQ‐GPX1000, CSTB2024NSCQ‐MSX0699. The Science and Technology Research Program of Chongqing Municipal Education Commission, KJZD‐K202500513. The earmarked fund for China Agriculture Research System, CARS‐44‐KXJ20. Chongqing Modern Agricultural Industry Technology System, COMAITS202516. Chongqing Graduate Student Research Innovation Project, CYS260411.

## Conflicts of Interest

The authors declare no conflicts of interest.

## Supporting information


**Figure S1:** AcSirt2 expression constructs and validation of anti‐senescence effects in H_2_O_2_‐treated cells.
**Figure S2:** Effects of AcSirt2 overexpression on senescence markers in mammalian cells.
**Figure S3:** Effects of AcSirt2 overexpression on mRNA levels of mitochondrial dynamics‐related genes in mammalian cells.
**Figure S4:** Effects of AcSirt2 overexpression on mRNA levels of mitophagy‐related genes in mammalian cells.
**Figure S5:** Synthesis of dsRNA for *AcSirt2*, specificity assessment, and analysis of downstream gene expression.
**Figure S6:** Effects of the Sirt2‐specific inhibitor AK‐1 on lifespan and locomotor ability in worker bees.
**Figure S7:** Quantification of SA‐β‐gal staining in worker bee brains from different treatment groups.
**Figure S8:** Effects of H_2_O_2_ concentration on viability and senescence in HaCaT and HEK‐293T cells.
**Figure S9:** Survival curves of *A. cerana* fed with pure 50% sucrose syrup or 50% sucrose syrup supplemented with DMSO at concentrations of 0.2%, 0.4%, 0.6%, 0.8%, and 1%.
**Table S1:** KEGG enrichment analysis of shared DEGs across worker bee differen age groups.
**Table S2:** Primer sequences used in this study.

## Data Availability

The data supporting this study are included in the paper and its Supporting Information—S1. Transcriptomic sequencing raw data derived from the head tissues of *A. cerana* worker bees have been deposited in the NCBI Sequence Read Archive (SRA) under accession number PRJNA1234368. Uncropped western blot images have been deposited in Figshare (https://doi.org/10.6084/m9.figshare.31627888). Additional data are available from the corresponding author upon reasonable request.
