# Supplementary material for: Unveiling the AcSirt2‐FOXO‐Mitophagy Axis: Insights Into Mitochondrial Quality Control and Delayed Aging in Apis cerana
Source: Aging Cell. 2026 Jul 26;25(8):e70645. doi: 10.1111/acel.70645 (PMC13401901; doi:10.1111/acel.70645)
Supplement: Supplementary file 1 — Figure S1: AcSirt2 expression constructs and validation of anti‐senescence effects in H2O2‐treated cells. Figure S2: Effects of AcSirt2 overexpression on senescence markers in mammalian cells. Figure S3: Effects of AcSirt2 overexpression on mRNA levels of mitochondrial dynamics‐related genes in mammalian cells. Figure S4: Effects of AcSirt2 overexpression on mRNA levels of mitophagy‐related genes in mammalian cells. Figure S5: Synthesis of dsRNA for AcSirt2, specificity assessment, and analysis of downstream gene expression. Figure S6: Effects of the Sirt2‐specific inhibitor AK‐1 on lifespan and locomotor ability in worker bees. Figure S7: Quantification of SA‐β‐gal staining in worker bee brains from different treatment groups. Figure S8: Effects of H2O2 concentration on viability and senescence in HaCaT and HEK‐293T cells. Figure S9: Survival curves of A. cerana fed with pure 50% sucrose syrup or 50% sucrose syrup supplemented with DMSO at concentrations of 0.2%, 0.4%, 0.6%, 0.8%, and 1%. Table S1: KEGG enrichment analysis of shared DEGs across worker bee differen age groups. Table S2: Primer sequences used in this study. [file ACEL-25-e70645-s001.docx]

**Supporting Information**

**Unveiling the AcSirt2-FOXO-mitophagy axis: insights into mitochondrial quality control and delayed aging in *Apis cerana***

*Qiang Ma, Zhengang Ma*, Tingyue Huang, Qianmin Hai, Xiaoqun Dang, Jinshan Xu, Jialing Bao, Zachary Y. Huang, Zeyang Zhou**


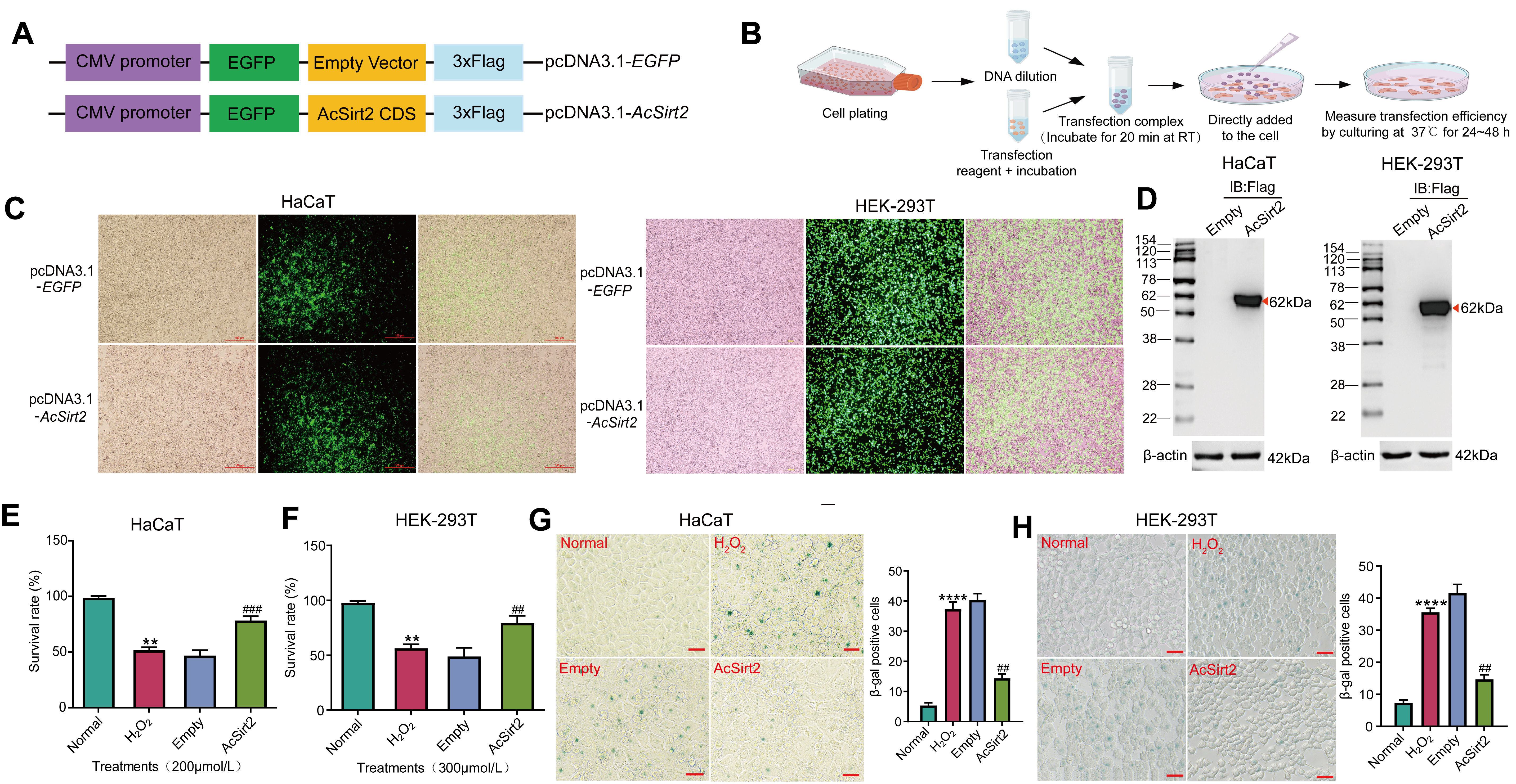


**FIGURE S1 |** **AcSirt2 expression constructs and validation of anti-senescence effects in H₂O₂-treated cells.** **(A)** Schematic diagram of the pcDNA3.1-*AcSirt2* recombinant expression plasmid. The plasmid carries an EGFP fluorescent tag, a 3 × Flag tag, and the AcSirt2 coding sequence; the multiple cloning site (MCS) indicates the insertion region for the target gene. **(B)** Schematic illustration of the cell transfection experimental workflow. **(C)** Fluorescence microscopy images showing EGFP expression in HaCaT and HEK-293T cells transfected with the AcSirt2 plasmid or empty vector. Scale bar: 100 µm (*n* = 3). **(D)** Western blot analysis of Flag-AcSirt2 protein expression in HaCaT and HEK-293T cells (*n* = 3). **(E, F)** Cell viability of HaCaT (E) and HEK-293T (F) cells measured by CCK-8 assay (*n* = 3). **(G, H)** Representative images and quantification of SA-β-gal staining in HaCaT (G) and HEK-293T (H) cells; blue-stained cells indicate SA-β-gal-positive senescent cells. Scale bar: 50 µm (*n* = 3). Data in (E-H) are presented as mean ± SEM. Statistical significance in (E-H) was determined by one-way ANOVA [*F* (3, 8)] with Tukey's post-hoc test: ^**^*p* < 0.01, ^****^*p* < 0.0001 compared with the normal group; ^##^*p* < 0.01, ^###^*p* < 0.001 compared with the H₂O₂-treated group. No significant differences were detected between the H₂O₂-treated group and the H₂O₂ + Empty vector group (*p* > 0.05).


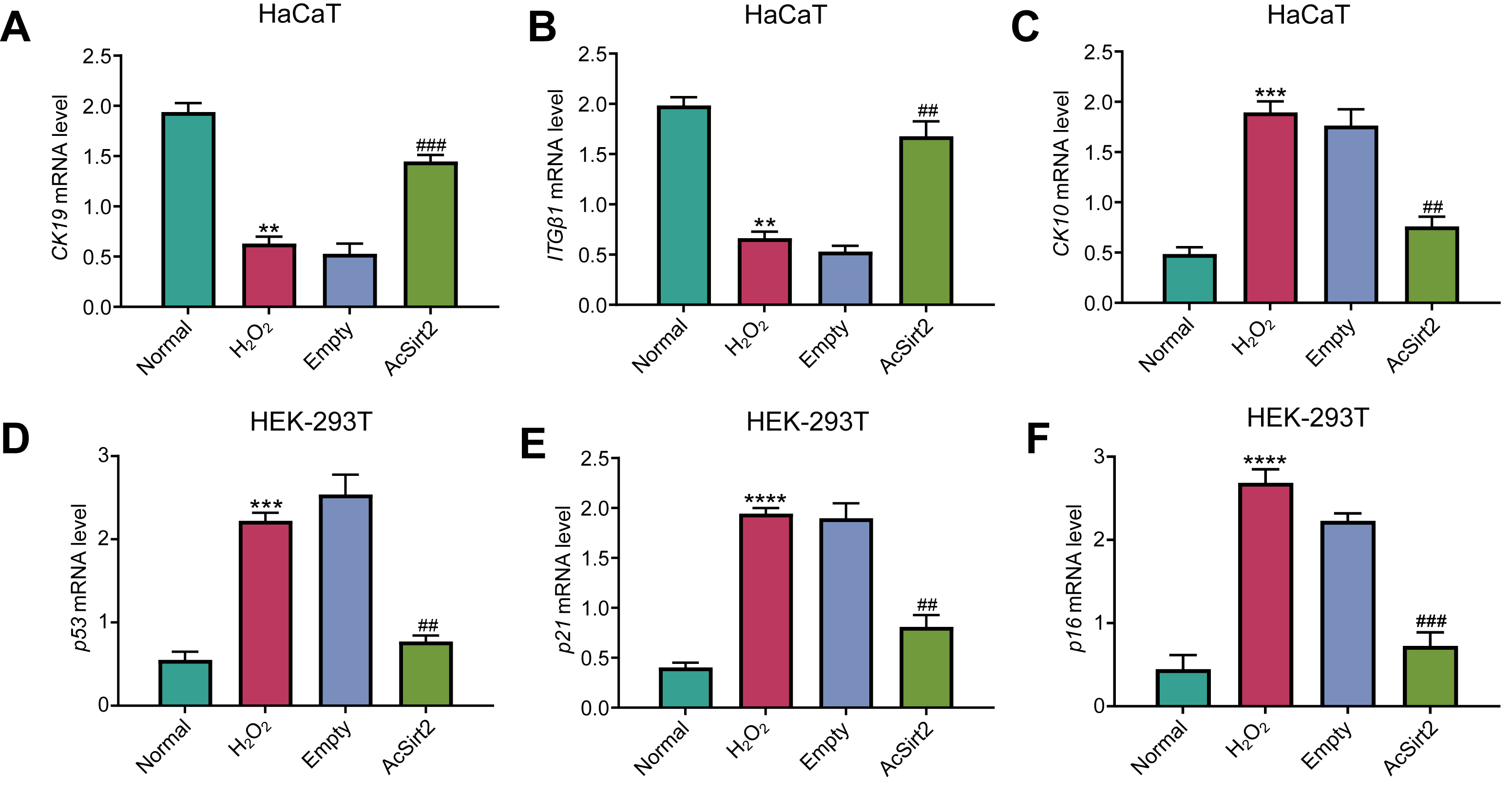


**FIGURE S2 | Effects of AcSirt2 overexpression on senescence markers in mammalian cells.** **(A-C)** Relative mRNA expression levels of the senescence genes *CK19* (A), *ITGβ1* (B), and the terminal differentiation gene *CK10* (C) in HaCaT cells (*n* = 3). **(D-F)** Relative mRNA expression levels of the senescence genes *p53*(D), *p21*(E), and *p16* (F) in HEK-293T cells (*n* = 3). All expressions were determined by RT-qPCR. Data are presented as mean ± SEM. Statistical significance was determined by one-way ANOVA [*F* (3, 8)] with Tukey's post-hoc test: ^**^*p* < 0.01, ^***^*p* < 0.001, ^****^*p* < 0.0001 *vs*. Normal group; ^##^*p* < 0.01, ^###^*p* < 0.001 *vs*. H₂O₂-treated group.


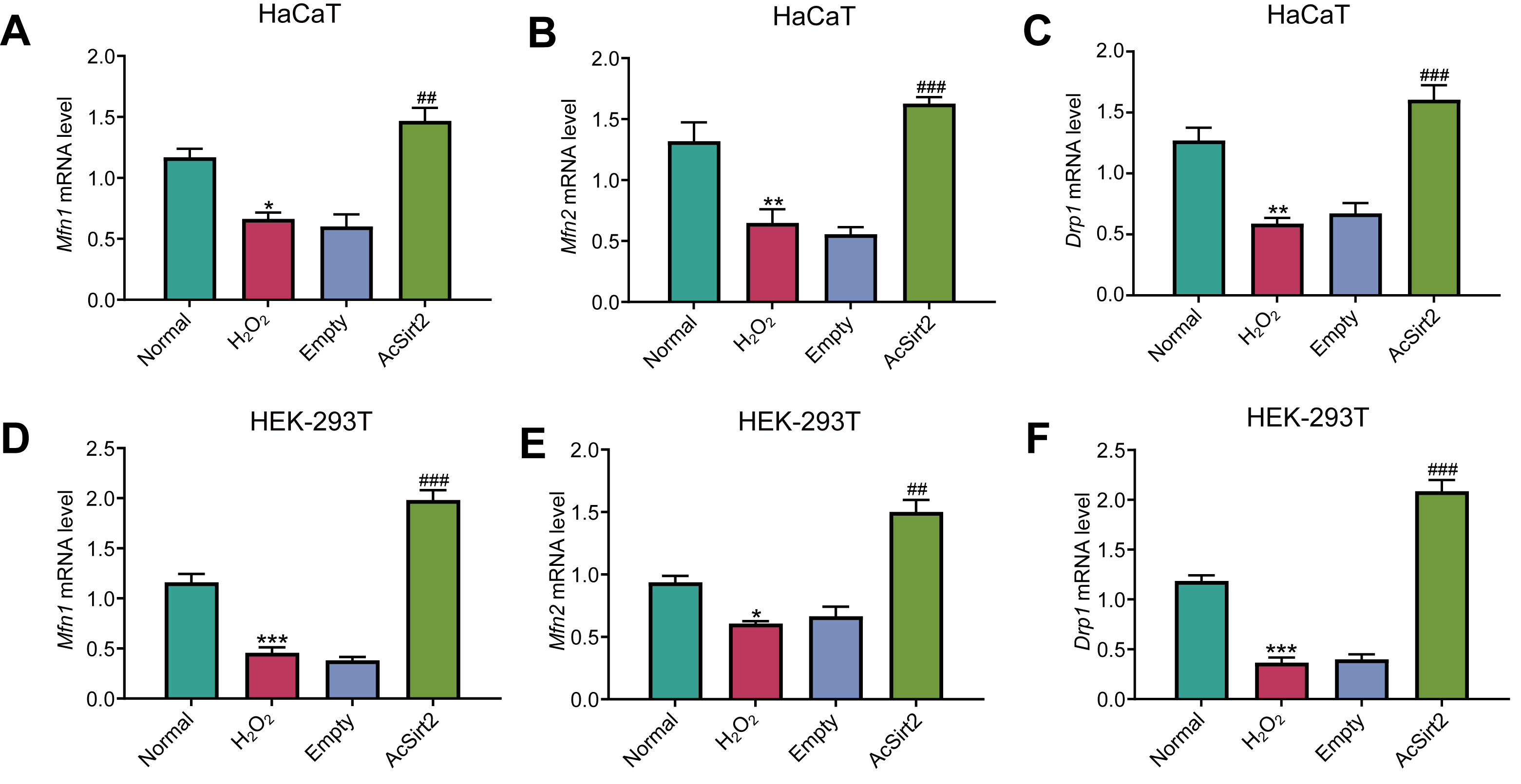


**FIGURE S3 | Effects of AcSirt2 overexpression on mRNA levels of mitochondrial dynamics-related genes in mammalian cells.** (**A-C)** Relative mRNA expression levels of the fusion genes *Mfn1* (A) and *Mfn2* (B), and the fission gene *Drp1* (C) in HaCaT cells (*n* = 3). **(D-F)** Relative mRNA expression levels of *Mfn1* (D), *Mfn2* (E), and *Drp1* (F) in HEK-293T cells (*n* = 3). Gene expression was analyzed by RT-qPCR. Data are presented as mean ± SEM. Statistical significance was determined by one-way ANOVA [*F* (3, 8)] with Tukey's post-hoc test: ^*^*p* < 0.05, ^**^*p* < 0.01, ^***^*p* < 0.001 *vs*. Normal group; ^##^*p* < 0.01, ^###^*p* < 0.001 *vs*. H₂O₂-treated group.


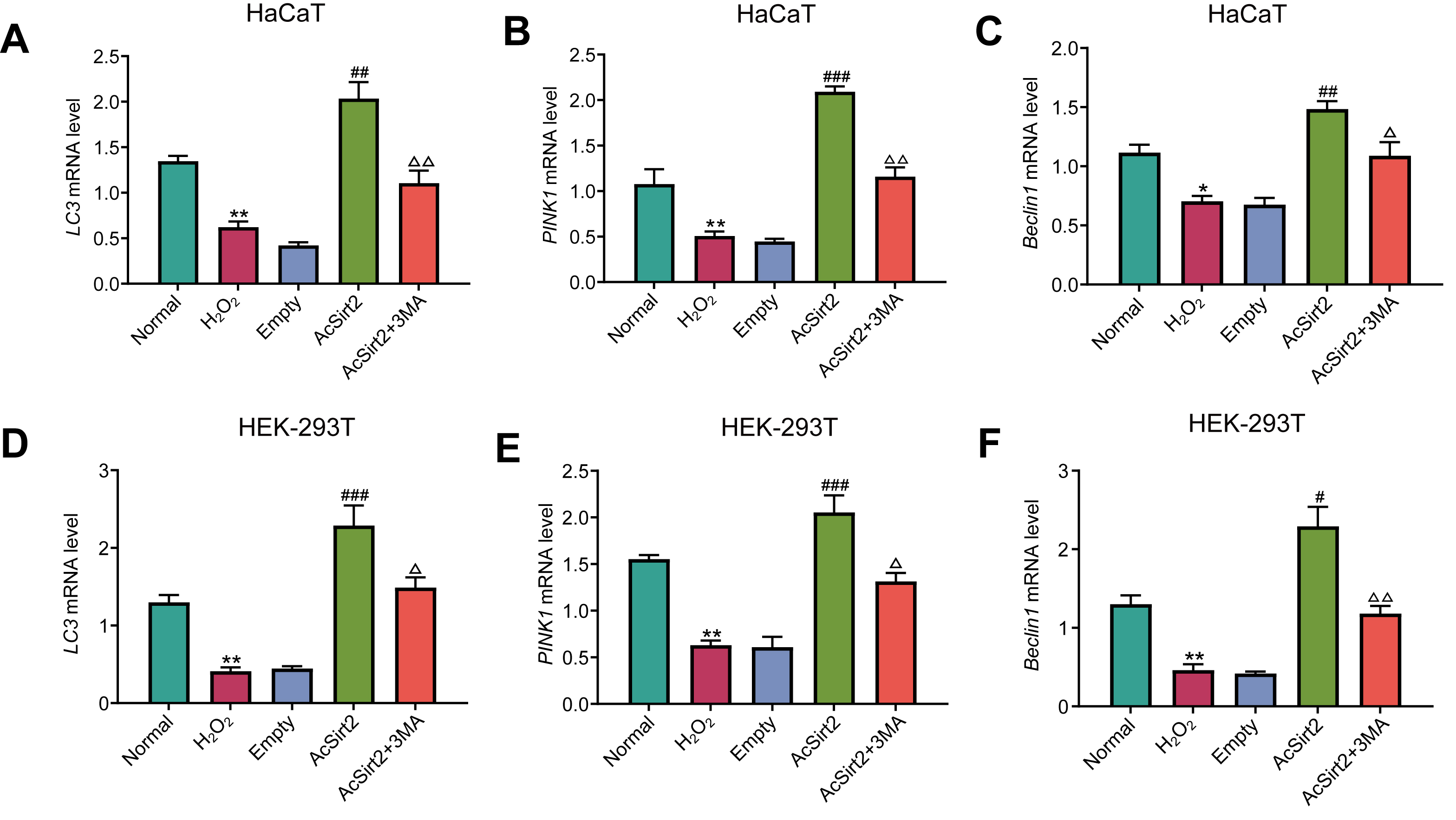


**FIGURE S4 | Effects of AcSirt2 overexpression on mRNA levels of mitophagy-related genes in mammalian cells.** **(A-C)** Relative mRNA expression levels of the mitophagy-related genes *LC3* (A), *PINK1* (B), and *Beclin1* (C) in HaCaT cells (*n* = 3). **(D-F)** Relative mRNA expression levels of *LC3* (D), *PINK1* (E), and *Beclin1* (F) in HEK-293T cells (*n* = 3). Gene expression was analyzed by RT-qPCR. Data are presented as mean ± SEM. Statistical significance was determined by one-way ANOVA [*F* (4, 10)] with Tukey's post-hoc test: ^*^*p* < 0.05, ^**^*p* < 0.01 *vs*. Normal group; ^#^*p* < 0.05, ^##^*p* < 0.01, ^###^*p* < 0.001 *vs*. H₂O₂-treated group; ^Δ^*p* < 0.05, ^ΔΔ^*p* < 0.01 *vs*. AcSirt2 transfection group.


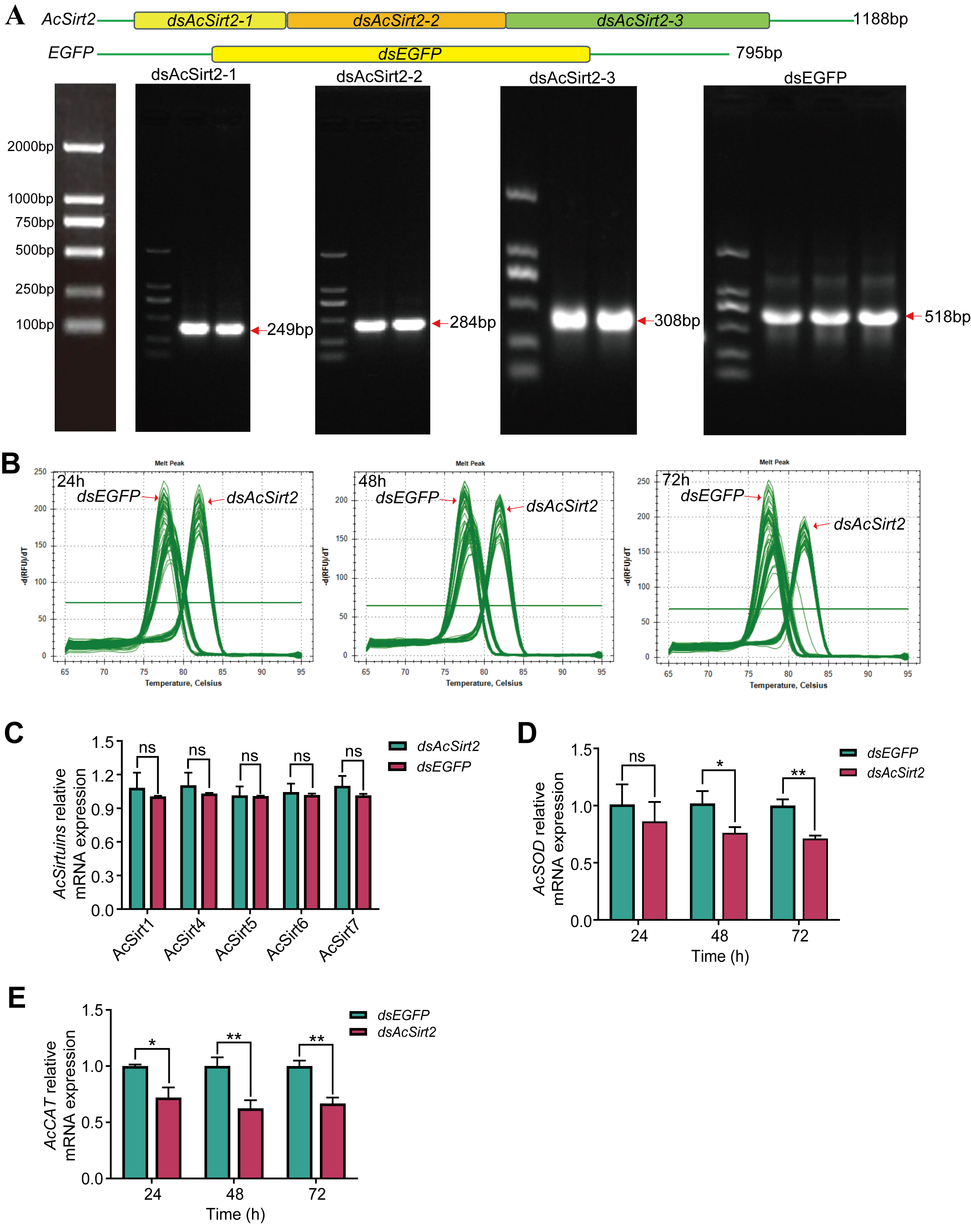


**FIGURE S5 | Synthesis of dsRNA for *AcSirt2*,** **specificity assessment*,* and analysis of downstream gene expression.** **(A)** Validation of *in vitro* synthesized dsRNA for different *AcSirt2* fragments (*dsAcSirt2-1*, *-2*, *-3*) and control *dsEGFP*. The expected amplification products of 249 bp, 284 bp, 308 bp, and 518 bp, respectively, confirm successful synthesis. Lane M: DNA molecular weight marker. **(B)** Melt curve analysis for RT-qPCR detection of *AcSirt2*. Both *dsAcSirt2* and *dsEGFP* control groups showed single, specific peaks at 24, 48, and 72 h post-dsRNA treatment, indicating high primer specificity and reliable quantification. **(C)** Specificity assessment of dsAcSirt2-mediated knockdown. Expression of *AcSirt1*, *AcSirt4*, *AcSirt5*, *AcSirt6*, and *AcSirt7* in the brain tissues of worker bees at 72 h after *dsAcSirt2* or *dsEGFP* treatment, measured by RT‑qPCR (*n* = 3). **(D, E)** Relative mRNA expression levels of the antioxidant-related genes *AcSOD* (C) and *AcCAT* (D) in the brain tissues of worker bees after knockdown with *dsAcSirt2* or *dsEGFP* (control) (*n* = 3 per treatment per time point). Data in (C-E) are presented as mean ± SEM. Statistical significance in (D, E) was determined by two-way ANOVA (treatment × time) with Tukey's post-hoc test: treatment *F* (1, 12), time *F* (2, 12), interaction *F* (2, 12); **p* < 0.05, ***p* < 0.01 *vs*. *dsEGFP* group at corresponding time point. No significant (ns) differences were detected between *dsAcSirt2* and *dsEGFP* groups for any non-target sirtuin in (C) (Student's t-test, *p* > 0.05).


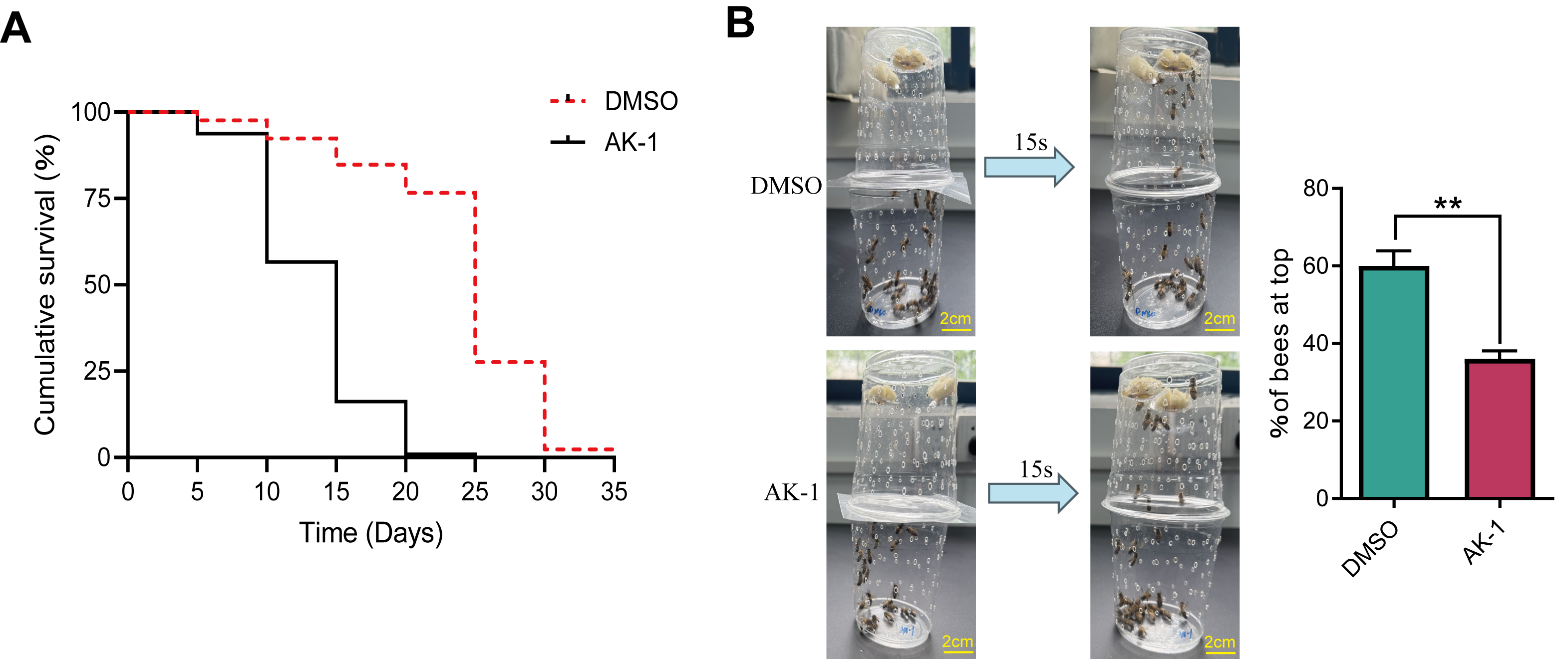


**FIGURE S6 |** **Effects of the Sirt2-specific inhibitor AK-1 on lifespan and locomotor ability in worker bees.** **(A)** Survival curves of worker bees continuously fed the Sirt2-specific inhibitor AK-1 or DMSO (control) from day 7 post-eclosion (*N* = 150 per group; 50 bees per cup, 3 biological replicates). AK-1 treatment significantly shortened the mean lifespan (DMSO: 31.7 ± 1.2 days; AK-1: 21.7 ± 2.9 days; log-rank (Mantel‑Cox) test, df = 1, χ² = 19.2, *p* < 0.0001). **(B)** The percentage of bees that successfully reached the top feeding platform within 15 s in the locomotor assay (*N* = 120 per group; 40 bees per cup, 3 biological replicates). AK-1 treated bees showed a significantly lower success rate than controls. Data are presented as mean ± SEM. Statistical significance was determined by the chi‑square test (df *=* 1). ^**^*p* < 0.01.


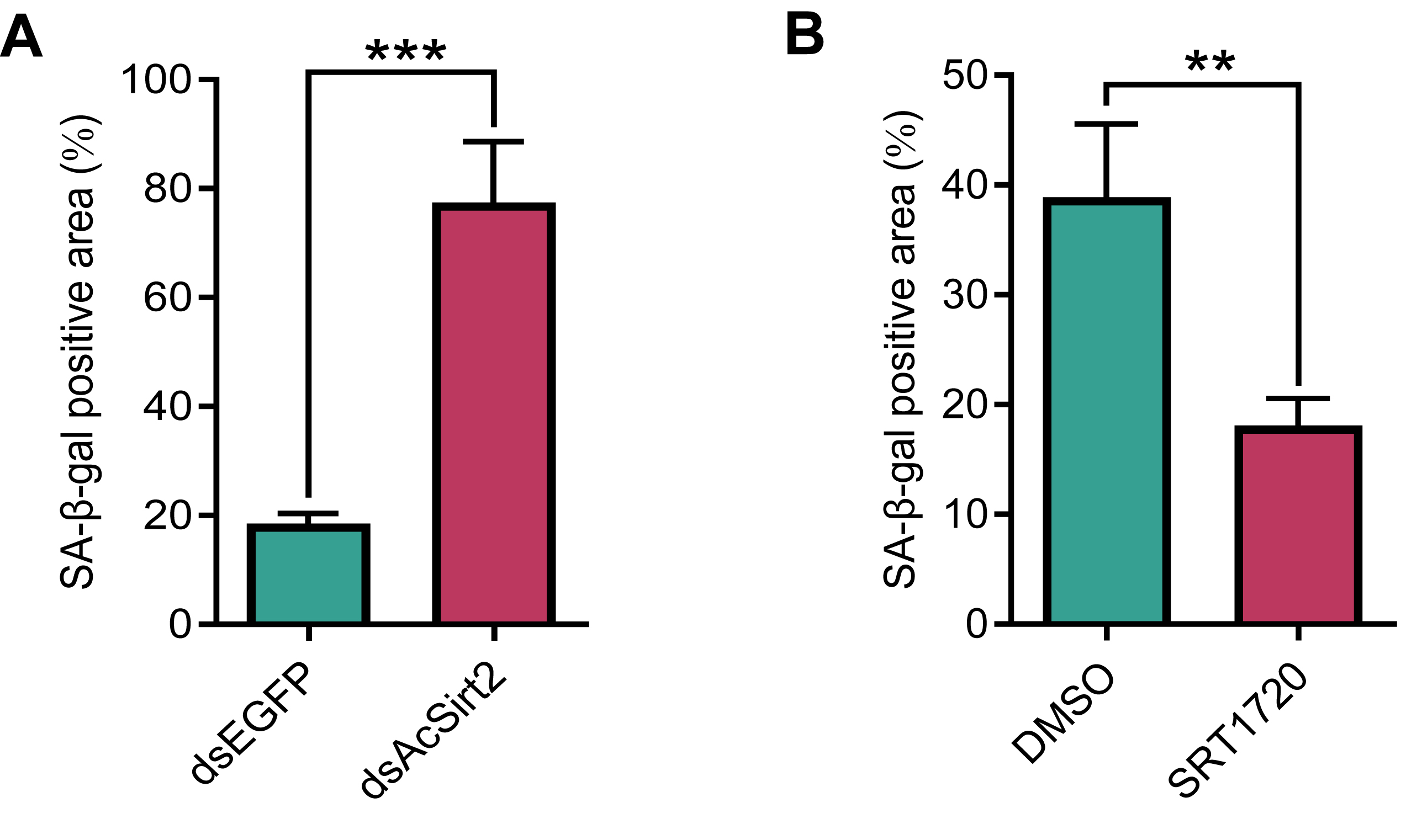


**FIGURE S7** | **Quantification of SA-β-gal staining in worker bee brains from different treatment groups.** **(A)** Percentage of SA-β-gal-positive area in brain tissue sections from *dsEGFP* and *dsAcSirt2* treatment groups. **(B)** Percentage of SA-β-gal-positive area in brain tissue sections from DMSO and SRT1720 treatment groups. Data are presented as mean ± SEM (*n* = 3 biological replicates, with five random fields analyzed per replicate). Statistical significance in (A) and (B) was determined by unpaired Student's *t*-test: ***p* < 0.01, ****p* < 0.001.


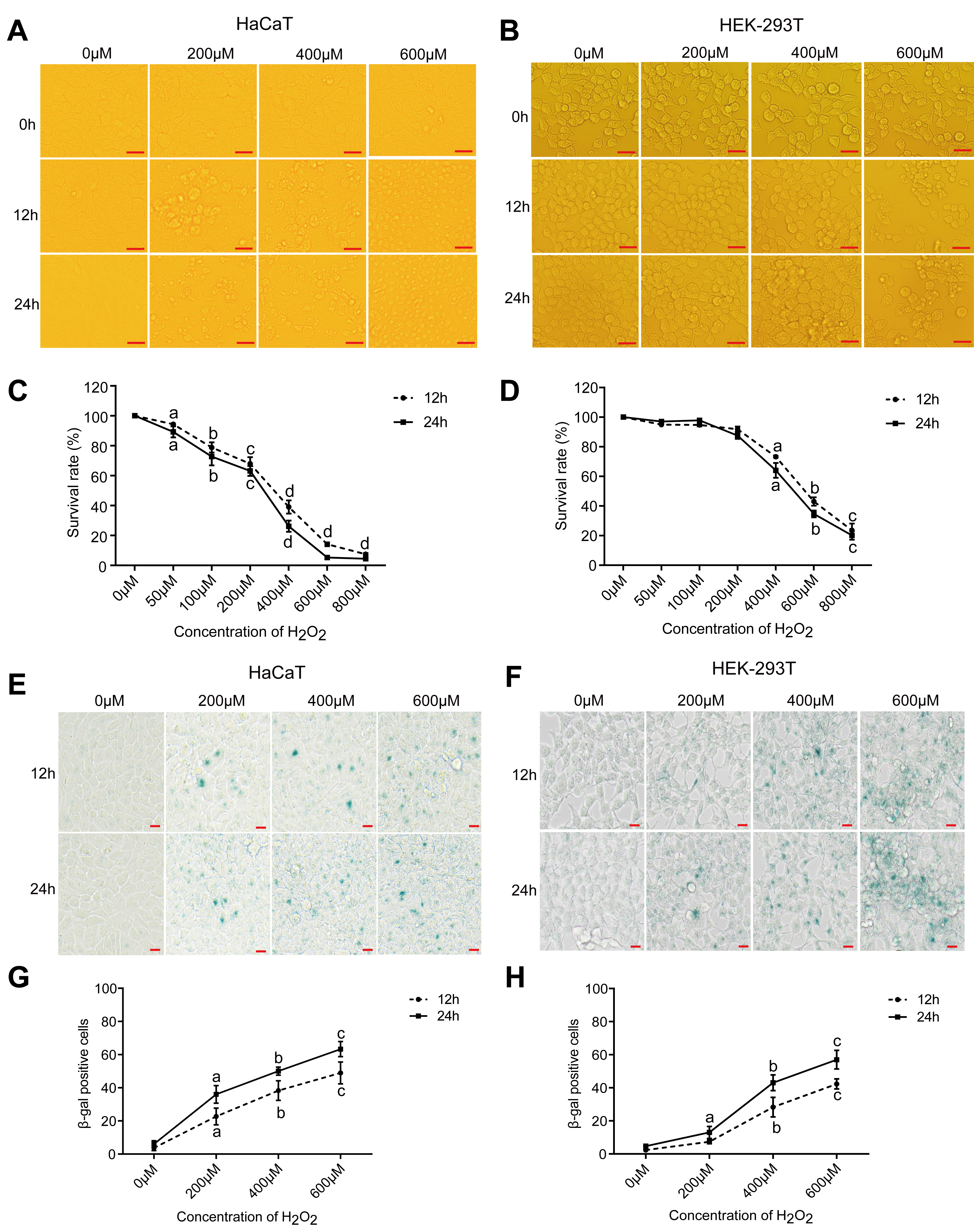


**FIGURE S8 | Effects of H₂O₂ concentration on viability and senescence in HaCaT and HEK-293T cells.** (**A, B)** Morphology of HaCaT (A) and HEK-293T (B) cells treated with different concentrations of H₂O₂ (*n* = 3). (**C, D)** Viability of HaCaT (C) and HEK-293T (D) cells after treatment with different concentrations of H₂O₂, measured by CCK-8 assay (*n* = 3). (**E, F)** SA-β-gal staining of HaCaT (E) and HEK-293T (F) cells treated with different concentrations of H₂O₂. (**G, H)** Quantification of SA-β-gal-positive HaCaT (G) and HEK-293T (H) cells (*n* = 3). Data in (C, D, G, H) are presented as mean ± SEM. Statistical analysis in (C, D, G, H) was performed by one-way ANOVA [*F* (7, 16)] with Tukey's post-hoc test. Different lowercase letters indicate statistically significant differences (*p* < 0.05).


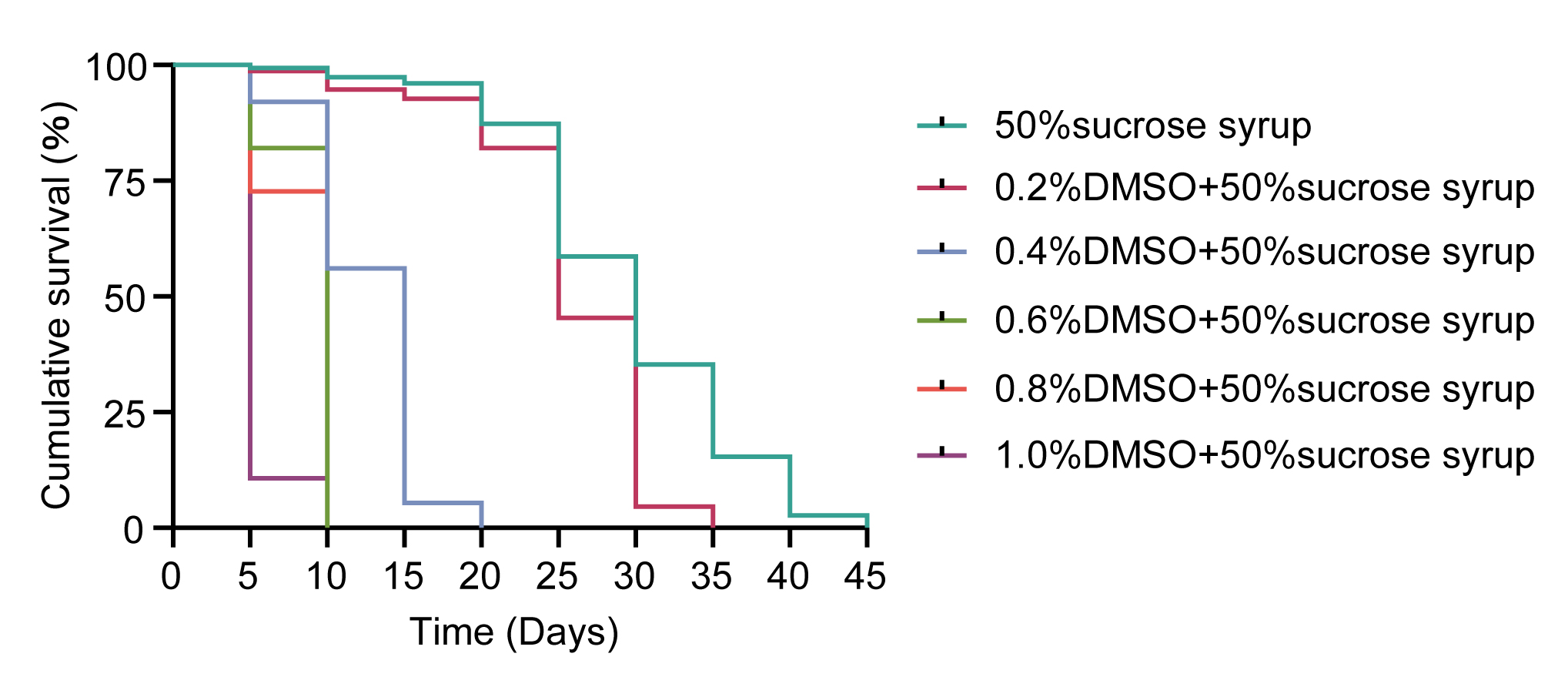


**FIGURE S9 |** **Survival curves of *A. cerana* fed with pure 50% sucrose syrup or 50% sucrose syrup supplemented with DMSO at concentrations of 0.2%, 0.4%, 0.6%, 0.8%, and 1%.** *N* = 150 per group; 50 bees per cup, with 3 biological replicates. Data were analyzed using the Log-rank (Mantel-Cox) test (df = 5).

**Table S1 |** KEGG enrichment analysis of shared DEGs across worker bee differen age groups

| **KEGGID** | **Description** | **GeneRatio** | **BgRatio** | **pvalue** | **padj** | **geneID** | **keggID** | **Count** |
| --- | --- | --- | --- | --- | --- | --- | --- | --- |
| ame04213 | Longevity regulating pathway - multiple species | 8/14 | 52/3184 | 5.08E-09 | 1.78E-07 | 107996027/107996026/107996022/108000803/107993802/107999690/108000922/107994298 | LOC107996027/LOC107996026/LOC107996022/LOC108000803/LOC107993802/LOC107999690/LOC108000922/LOC107994298 | ame:724488/ame:724274/ame:724367/ame:411917/ame:443552/ame:724442/ame:410812/ame:551668 |
| ame04068 | FoxO signaling pathway | 6/14 | 60/3184 | 7.56E-06 | 0.00013233 | 108000803/107993802/107999690/108000922/108002938/107998969 | LOC108000803/LOC107993802/LOC107999690/LOC108000922/LOC108002938/LOC107998969 | ame:411917/ame:443552/ame:724442/ame:410812/ame:550739/ame:552718 |
| ame00020 | Citrate cycle (TCA cycle) | 5/14 | 39/3184 | 1.40E-05 | 0.000163816 | 108000164/107998211/108003960/107993356/108000656 | LOC108000164/LOC107998211/LOC108003960/LOC107993356/LOC108000656 | ame:408734/ame:551169/ame:409155/ame:551958/ame:550686 |
| ame04142 | Lysosome | 5/14 | 91/3184 | 0.000838137 | 0.007333697 | 107997586/108003248/107996942/107997817/108000398 | LOC107997586/LOC108003248/LOC107996942/LOC107997817/LOC108000398 | ame:724386/ame:409708/ame:727146/ame:408851/ame:413119 |
| ame01200 | Carbon metabolism | 5/14 | 106/3184 | 0.001673451 | 0.011714156 | 107993802/108000164/107998211/108003960/107993356 | LOC107993802/LOC108000164/LOC107998211/LOC108003960/LOC107993356 | ame:443552/ame:408734/ame:551169/ame:409155/ame:551958 |
| ame04214 | Apoptosis - fly | 3/14 | 52/3184 | 0.009116148 | 0.053177533 | 108000922/108001217/107995837 | LOC108000922/LOC108001217/LOC107995837 | ame:410812/ame:410226/ame:410263 |
| ame00380 | Tryptophan metabolism | 2/14 | 23/3184 | 0.015702177 | 0.073267126 | 107993802/108003960 | LOC107993802/LOC108003960 | ame:443552/ame:409155 |
| ame04137 | Mitophagy - animal | 3/14 | 65/3184 | 0.016746772 | 0.073267126 | 108000922/108001217/107995837 | LOC108000922/LOC108001217/LOC107995837 | ame:410812/ame:410226/ame:410263 |
| ame04136 | Autophagy - other | 2/14 | 26/3184 | 0.019858502 | 0.075116064 | 107994298/108004280 | LOC107994298/LOC108004280 | ame:551668/ame:726562 |
| ame00600 | Sphingolipid metabolism | 2/14 | 28/3184 | 0.02285823 | 0.075116064 | 108003248/107996942 | LOC108003248/LOC107996942 | ame:409708/ame:727146 |
| ame04141 | Protein processing in endoplasmic reticulum | 4/14 | 132/3184 | 0.023607906 | 0.075116064 | 107996027/107996026/107996022/108001217 | LOC107996027/LOC107996026/LOC107996022/LOC108001217 | ame:724488/ame:724274/ame:724367/ame:410226 |
| ame04320 | Dorso-ventral axis formation | 2/14 | 30/3184 | 0.026033462 | 0.07593093 | 108000922/107998969 | LOC108000922/LOC107998969 | ame:410812/ame:552718 |
| ame04150 | mTOR signaling pathway | 3/14 | 91/3184 | 0.040258919 | 0.108389396 | 108000922/107994298/107998969 | LOC108000922/LOC107994298/LOC107998969 | ame:410812/ame:551668/ame:552718 |
| ame04140 | Autophagy - animal | 1/14 | 112/3184 | 0.067179464 | 0.167948659 | 108000922/107994298/108004280 | LOC108000922/LOC107994298/LOC108004280 | ame:410812/ame:551668/ame:726562 |
| ame04146 | Peroxisome | 1/14 | 68/3184 | 0.11214148 | 0.261663453 | 107993802/108003267 | LOC107993802/LOC108003267 | ame:443552/ame:412986 |
| ame00531 | Glycosaminoglycan degradation | 1/14 | 16/3184 | 0.127663745 | 0.279264443 | 108000398 | LOC108000398 | ame:413119 |
| ame00511 | Other glycan degradation | 1/14 | 18/3184 | 0.142472077 | 0.280610494 | 108003248 | LOC108003248 | ame:409708 |
| ame00760 | Nicotinate and nicotinamide metabolism | 1/14 | 21/3184 | 0.164231604 | 0.280610494 | 108000803 | LOC108000803 | ame:411917 |
| ame00190 | Oxidative phosphorylation | 2/14 | 88/3184 | 0.170294735 | 0.280610494 | 108000164/107998211 | LOC108000164/LOC107998211 | ame:408734/ame:551169 |
| ame04013 | MAPK signaling pathway - fly | 2/14 | 88/3184 | 0.170294735 | 0.280610494 | 108000922/107998969 | LOC108000922/LOC107998969 | ame:410812/ame:552718 |
| ame00982 | Drug metabolism - cytochrome P450 | 1/14 | 22/3184 | 0.17136589 | 0.280610494 | 107997421 | LOC107997421 | ame:409490 |
| ame00980 | Metabolism of xenobiotics by cytochrome P450 | 1/14 | 24/3184 | 0.185458938 | 0.280610494 | 107997421 | LOC107997421 | ame:409490 |
| ame00062 | Fatty acid elongation | 1/14 | 25/3184 | 0.192418625 | 0.280610494 | 108000515 | LOC108000515 | ame:724552 |
| ame00785 | Lipoic acid metabolism | 1/14 | 25/3184 | 0.192418625 | 0.280610494 | 108003960 | LOC108003960 | ame:409155 |
| ame00630 | Glyoxylate and dicarboxylate metabolism | 1/14 | 29/3184 | 0.219689073 | 0.307564703 | 107993802 | LOC107993802 | ame:443552 |
| ame01040 | Biosynthesis of unsaturated fatty acids | 1/14 | 32/3184 | 0.239557704 | 0.315664797 | 108000515 | LOC108000515 | ame:724552 |
| ame00640 | Propanoate metabolism | 1/14 | 34/3184 | 0.252531837 | 0.315664797 | 107993356 | LOC107993356 | ame:551958 |
| ame01210 | 2-Oxocarboxylic acid metabolism | 1/14 | 34/3184 | 0.252531837 | 0.315664797 | 108003960 | LOC108003960 | ame:409155 |
| ame00480 | Glutathione metabolism | 1/14 | 38/3184 | 0.277843601 | 0.320691884 | 107997421 | LOC107997421 | ame:409490 |
| ame00983 | Drug metabolism - other enzymes | 1/14 | 38/3184 | 0.277843601 | 0.320691884 | 107997421 | LOC107997421 | ame:409490 |
| ame00310 | Lysine degradation | 1/14 | 39/3184 | 0.284041383 | 0.320691884 | 108003960 | LOC108003960 | ame:409155 |
| ame04624 | Toll and Imd signaling pathway | 1/14 | 49/3184 | 0.343267344 | 0.375448658 | 107995531 | LOC107995531 | ame:725541 |
| ame01212 | Fatty acid metabolism | 1/14 | 56/3184 | 0.381887454 | 0.405032148 | 108000515 | LOC108000515 | ame:724552 |
| ame04148 | Efferocytosis | 1/14 | 69/3184 | 0.447886587 | 0.461059722 | 108000803 | LOC108000803 | ame:411917 |
| ame04310 | Wnt signaling pathway | 1/14 | 91/3184 | 0.544415763 | 0.544415763 | 108000803 | LOC108000803 | ame:411917 |

**Table S****2** **|** Primer sequences used in this study

| **Gene** | **Primer sequence** | **Amplicon Size (bp)** | **Purpose** |
| --- | --- | --- | --- |
| *Hs**Mfn1* | F: GTTACCGAGGAGGTGGCAAA | 216 | qPCR |
|  | R: GGTCTGAAGCACTAAGGCGT |  |  |
| *HsMfn2* | F: GGACCCCGTTACCACAGAAG | 244 | qPCR |
|  | R: AAGGAGAAAGGCCTCATGGC |  |  |
| *HsDrp1* | F: AGAAAATGGGGTGGAAGCAGA | 220 | qPCR |
|  | R: AGGCACCTTGGTCATTCCTG |  |  |
| *HsBeclin1* | F: CCACAGAAAGTGCCAACAGC | 194 | qPCR |
|  | R: GACGTTGAGCTGAGTGTCCA |  |  |
| *HsLC3* | F: CGCTACAAGGGTGAGAAGCA | 256 | qPCR |
|  | R: AGAAGCCGAAGGTTTCCTGG |  |  |
| *HsPINK1* | F: CTGGGCCTCATCGAGGAAAA | 175 | qPCR |
|  | R: AGCCCTTACCAATGGACTGC |  |  |
| *HsCK10* | F: GGCAAAATCAAGGAGTGGTATG | 142 | qPCR |
|  | R: GAAGCAGGATGTTGGCATTATC |  |  |
| *HsITGβ1* | F: TCGGGACAAATTACCCCAGC | 119 | qPCR |
|  | R: CCATGACCTCGTTGTTCCCA |  |  |
| *HsCK19* | F: GCCACTACTACACGACCATCCA | 142 | qPCR |
|  | R: AGAGCCTGTTCCGTCTCAAACT |  |  |
| *Hsp53* | F: TGTGACTTGCACGTACTCCC | 199 | qPCR |
|  | R: ACCATCGCTATCTGAGCAGC |  |  |
| *Hsp21* | F: GCGACTGTGATGCGCTAATG | 141 | qPCR |
|  | R: GAAGGTAGAGCTTGGGCAGG |  |  |
| *Hsp16* | F: AGGTCATGATGATGGGCAGC | 321 | qPCR |
|  | R: AATCGGGGATGTCTGAGGGA |  |  |
| *HsActin* | F: TTCCTTCCTGGGCATGGAGT | 265 | qPCR |
|  | R: TCTTCATTGTGCTGGGTGCC |  |  |
| *AcSirt2* | F: GCACCATATACTCTTCCATGGA | 221 | qPCR |
|  | R: TCCACTAGAGATGCAAAAGGTT |  |  |
| *AcSOD* | F: AGCACCATGCGACTTATGTGA | 193 | qPCR |
|  | R: AGGAGTGCAGCATCTGGTTT |  |  |
| *AcCAT* | F: TTCCGGTCATTCGACCGTTT | 181 | qPCR |
|  | R: AATCGACGAAAAGCCAGGGT |  |  |
| *AcActin* | F: TCCTGCTATGTATGTCGC | 301 | qPCR |
|  | R: GGTTGCCATTTCCTGTTC |  |  |
| *pcDNA3.1-AcSirt2* | F: GCTGGATATCTGCAGAATTCATGTCTGAACATAATGAAGACACAGA | 1188 | Transfection |
|  | R: TAGTCACTTAAGCTTGGTACTTCTCTATTTAAACGTTCATATTCTCTTTTTATAAG |  |  |
| *dsAcSirt2-1* | F: TAATACGACTCACTATAGGGGGAGATAGAAGAATCTGATATGGAA | 249 | dsRNA Synthesis |
|  | R: TAATACGACTCACTATAGGGTTGTGATAAAGACCACTTGTTGGTG |  |  |
| *dsAcSirt2-2* | F: TAATACGACTCACTATAGGGGGCCGATGTCTTAAATGTAGA | 284 | dsRNA Synthesis |
|  | R: TAATACGACTCACTATAGGGTCTTTATTGATAAGTAAACGCGG |  |  |
| *dsAcSirt2-3* | F: TAATACGACTCACTATAGGGCACCAACAAGTGGTCTTTATCACAA | 308 | dsRNA Synthesis |
|  | R: TAATACGACTCACTATAGGGTCTACATTTAAGACATCGGCC |  |  |
| *dsEGFP* | F: TAATACGACTCACTATAGGGCGACAAGCAGAAGAACGGC | 518 | dsRNA Synthesis |
|  | R: TAATACGACTCACTATAGGGCGGTCTTGTAGTTGCCGTCG |  |  |

Note: *Actin* is reference gene.
